# Highly Sensitive, Ultrafast, and Broadband Photo‐Detecting Field‐Effect Transistor with Transition‐Metal Dichalcogenide van der Waals Heterostructures of MoTe_2_ and PdSe_2_

**DOI:** 10.1002/advs.202003713

**Published:** 2021-03-16

**Authors:** Amir Muhammad Afzal, Muhammad Zahir Iqbal, Ghulam Dastgeer, Aqrab ul Ahmad, Byoungchoo Park

**Affiliations:** ^1^ Department of Electrical and Biological Physics Kwangwoon University Wolgye‐Dong Seoul 01897 South Korea; ^2^ Nanotechnology Research Laboratory, Faculty of Engineering Sciences GIK Institute of Engineering Sciences and Technology Topi Khyber Pakhtunkhwa 23640 Pakistan; ^3^ School of Physics Peking University Beijing 100871 China; ^4^ IBS Center for Integrated Nanostructure Physics Sungkyunkwan University Suwon 16419 South Korea; ^5^ School of Physics and School of Microelectronics Dalian University of Technology Dalian 116000 China

**Keywords:** charge‐transfer transition, field‐effect transistors, photoresponsivity, specific detectivity, transition‐metal dichalcogenides

## Abstract

Recently, van der Waals heterostructures (vdWHs) based on transition‐metal dichalcogenides (TMDs) have attracted significant attention owing to their superior capabilities and multiple functionalities. Herein, a novel vdWH field‐effect transistor (FET) composed of molybdenum ditelluride (MoTe_2_) and palladium diselenide (PdSe_2_) is studied for highly sensitive photodetection performance in the broad visible and near‐infrared (VNIR) region. A high rectification ratio of 6.3 × 10^5^ is obtained, stemming from the sharp interface and low Schottky barriers of the MoTe_2_/PdSe_2_ vdWHs. It is also successfully demonstrated that the vdWH FET exhibits highly sensitive photo‐detecting abilities, such as noticeably high photoresponsivity (1.24 × 10^5^ A W^−1^), specific detectivity (2.42 × 10^14^ Jones), and good external quantum efficiency (3.5 × 10^6^), not only due to the intra‐TMD band‐to‐band transition but also due to the inter‐TMD charge transfer (CT) transition. Further, rapid rise (16.1 µs) and decay (31.1 µs) times are obtained under incident light with a wavelength of 2000 nm due to the CT transition, representing an outcome one order of magnitude faster than values currently in the literature. Such TMD‐based vdWH FETs would improve the photo‐gating characteristics and provide a platform for the realization of a highly sensitive photodetector in the broad VNIR region.

## Graphical abstract

Novel van der Waals heterostructure field‐effect transistors composed of transition‐metal dichalcogenides of molybdenum ditelluride (MoTe_2_) and palladium diselenide (PdSe_2_) are fabricated to realize highly sensitive photo‐detecting performance in the broad visible and near‐infrared region. Due to the charge‐transfer transition between MoTe_2_ and PdSe_2_, excellent photoresponse performance of the transistor is successfully realized together with rapid responses.

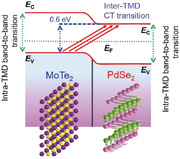

## 1 Introduction

Highly sensitive photodetectors based on semiconducting materials as light‐absorbing and charge‐transporting materials in either the visible and infrared (IR) spectral ranges are extensively investigated in relation to environment monitoring, thermal imaging, gas sensing, and imaging optoelectronic devices due to their small volumes and compatibility with on‐chip integration processes.^[^ ^1^ ^]^ For example, silicon‐based metal‐oxide‐semiconductor (Si‐MOS) field‐effect transistor (FET) photodetectors have attracted the interest of the semiconductor industry over the past few decades. However, given the continuous decrease in their size and the exponential increase in the density of the transistors, the performance capabilities of Si‐MOSFETs have started to degrade due to short channel effects, thus limiting further size reductions.^[^ ^2^ ^]^

More recently, graphene, a representative 2D material, has attracted significant attention owing to its fascinating electronic, thermal, and mechanical properties.^[^ ^3^ ^]^ Its ultrahigh charge carrier mobility (>200 000 cm^2^ V^−1^s^−1^) offers the potential for developing fast electronic devices.^[^ ^3^ ^]^ Ultrahigh mobility, however, arises from its zero‐bandgap characteristic, leading to low ON/OFF current ratios of graphene‐based transistors, which limit the wider application of this material.^[^ ^4^ ^]^ Thus, it is necessary to obtain a new 2D material with a wide tunable bandgap so as to realize multi‐functional nanoscale optoelectronic devices.^[^ ^5^ ^]^ Such a requirement has led researchers to investigate various transition‐metal dichalcogenides (TMDs) and other 2D materials in addition to graphene.^[^ ^6^ ^]^ Among them, black phosphorus (BP), an intrinsically p‐type TMD material, has been investigated due to its high hole mobility and ON/OFF current ratio. However, BP degrades easily under ambient air conditions, limiting its prospects in practical applications.^[^ ^7^ ^]^

Recently, molybdenum ditelluride (MoTe_2_), another p‐type TMD semiconductor material, has attracted the attention of researchers owing to its remarkable electrical and optical properties. MoTe_2_ has an indirect bandgap of 0.88 eV in a high‐quality multilayered bulk form, while it also has a direct optical bandgap of 1.10 eV in a monolayer structure.^[^ ^8^ ^]^ It has thus tremendous potential for use in the construction of p‐channel MOSFETs and for integration with MoS_2_ n‐channel MOSFETs, but it has limited applications in near‐infrared (NIR) photodetection due to its broad bandgap.^[^ ^8^, ^9^ ^]^ To overcome such constraints and to promote the high performance of MoTe_2_ in optoelectronics, several different approaches have been investigated.^[^ ^10^ ^]^ In relation to this, Xie et al. introduced a BP/MoTe_2_ junction to improve the photocurrent characteristics and observed relatively small values of the photoresponsivity (*R* ≈0.2 A W^−1^) and external quantum efficiency (*EQE* ≈ 48.1%) with a slow response time (≈2 ms). These small values may be caused by BP, which is a critical material that easily oxidizes.^[^ ^11^ ^]^

Recent advances have seen the discovery of another noble TMD semiconductor material, monochalcogenide palladium diselenide (PdSe_2_), which has potential applications for IR photodetection due to its small bandgap.^[^ ^12^ ^]^ PdSe_2_ exhibits a strong thickness‐dependent bandgap of 1.4–0 eV, with a monolayer of PdSe_2_ having a bandgap of ≈1.4 eV as well as bulk PdSe_2_, which is metallic, having a bandgap approaching 0 eV.^[^ ^13^ ^]^ Specifically, it has a pentagonal puckered morphology which promotes stability in air and stable performance.^[^ ^14^ ^]^ PdSe_2_ also has many unique electrical, photocurrent, and thermal characteristics as compared to conventional 2D graphene and other TMD materials.^[^ ^13^, ^15^ ^]^ Thus, it is considered to be an extraordinary material for optoelectronic applications in the visible and NIR regions owing to its high mobility with a narrow and tunable bandgap.^[^ ^16^ ^]^

Meanwhile, to fabricate efficacious electronic and optoelectronic devices, an exceptional approach related to van der Waals heterostructures (vdWHs) based on TMD semiconductors has been devised.^[^ ^17^ ^]^ Emerging devices based on TMD vdWHs have been successfully implemented in FETs,^[^ ^18^ ^]^ gas sensors,^[^ ^19^ ^]^ memory devices,^[^ ^20^ ^]^ high‐speed integrated circuits,^[^ ^21^ ^]^ energy storage devices,^[^ ^22^ ^]^ diodes,^[^ ^23^ ^]^ inverters,^[^ ^23a^ ^]^ negative differential resistance components,^[^ ^23a^ ^]^ amplifiers,^[^ ^24^ ^]^ spin‐FETs,^[^ ^25^ ^]^ and in water splitting.^[^ ^26^ ^]^ Further, this compact system of TMD vdWHs offers a new paradigm in which to overcome the limitations of conventional MOS heterostructures,^[^ ^17^ ^]^ especially for the engineering of state‐of‐the‐art (opto)electronic devices such as (photo‐) FETs, (optical) sensors, (photo‐) MOSFETs, and photodetectors that operate in the visible‐near‐infrared (VNIR) range (400–1000 nm). TMD vdWHs p‐n diodes are preferred over TMD homojunction‐based p‐n diodes owing to their high performance capabilities. Various methods have been used for the fabrication of TMD homojunction p‐n diodes, including chemical doping for Fermi‐level pinning, electrostatic doping to regulate the density of the carriers, and the creation of specific metal contacts to fabricate p‐type semiconductors. All of these methods deteriorate the performance capabilities of devices and degrade the charge carrier density.^[^ ^7^, ^27^, ^28^ ^]^

In such vdWH (opto)electronic devices, the photocurrent characteristics are highly enhanced up to a certain level owing to their sharp interfaces, tunneling mechanism, and band alignments.^[^ ^23a^ ^]^ However, large Schottky contact barrier heights (*φ*_B_s) between the metal contact and the TMD material (metal‐TMD heterojunction) obstruct the transport of photo‐excited charge carriers, which leads to the suppression of photocurrent generation.^[^ ^23^, ^27^ ^]^ From this perspective, the role of *φ*_B_ at the metal‐TMD junction should be emphasized to enhance the photocurrent characteristics. Hence, the successful use of TMD vdWHs as high‐performance VNIR photodetectors with highly improved characteristics, such as the photoresponsivity *R* and specific detectivity (D*) at a longer wavelength (*λ* > 900 nm), has yet to be realized.

In this work, we demonstrate for the first time a novel p‐MoTe_2_/n‐PdSe_2_ vdWH FET for highly sensitive and broadband photo‐detecting performance. A large rectification ratio (*RR*) of 6.3 × 10^5^ is achieved through the formation of Ohmic contact. Further, the photocurrent properties of the MoTe_2_/PdSe_2_ FET were studied under incident laser light with different input powers in a broad VNIR region (*λ* = 405–2000 nm). The key parameters of the photodetectors, in this case *R*, D*, *EQE*, and the response times, which can be tuned by applying back‐gate voltage (*V*_bg_) and explained based on the optical transition in terms of not only the intra‐TMD band‐to‐band transition but also inter‐TMD charge‐transfer (CT) transition, were also evaluated. Such high gate‐modulated photo‐detecting characteristics with the rapid response times of the p‐MoTe_2_/n‐PdSe_2_ vdWH FET devices are clear evidence of the excellent potential of optoelectronics and will likely be essential when developing highly efficient photodetectors in the broad VNIR region.

## 2 Results and Discussion

**Figure 1:**
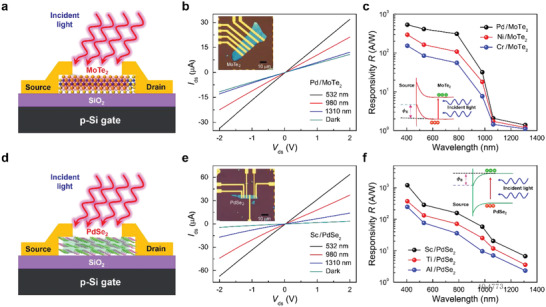
a) Schematic configuration of a p‐MoTe_2_ FET in contact with metal electrodes. b) *I*_ds_ **–** *V*_ds_ curves of a p‐MoTe_2_ FET with Pd electrodes in the dark and under the illumination of incident light with different wavelengths at a fixed input power of 20 nW at zero gate voltage. The inset shows an optical image of p‐MoTe_2_ FETs with Pd, Ni, and Cr electrodes. c) Responsivities (*R*s) of the p‐MoTe_2_ FETs with three different electrodes as a function of the wavelength of incident light at *V*_bg_ = 0 V. The inset shows a schematic energy band diagram of the photocurrent flows. d) Schematic diagram of an n‐PdSe_2_ FET in contact with metal electrodes. e) *I*_ds_–*V*_ds_ curves of the PdSe_2_ FET with Sc in the dark and under incident laser light with several different *λ*s at *V*_bg_ = 0 V. The inset shows an optical image of n‐PdSe_2_ FETs with Sc, Ti, and Al electrodes. f) Wavelength dependence of the responsivities *R*s for PdSe_2_ FETs with three different metal electrodes at *V*_bg_ = 0 V. The inset shows a schematic energy band diagram of the photocurrent flows.

Initially, prior to the investigation of the p‐MoTe_2_/n‐PdSe_2_ vdWH FET, we undertook electrical measurements of a p‐type MoTe_2_ FET and an n‐type PdSe_2_ FET separately with various metal electrodes in the dark and under incident light. **Figure** **1**a shows a schematic illustration of a p‐MoTe_2_ FET with metal electrodes (drain and source) under incident light. In order to investigate the roles of the metal electrodes on the device performance of the p‐MoTe_2_ FET, three different metals, here Pd, Ni, and Cr, having different work functions (*φ*s) were deposited as electrodes with a thickness of 6 nm onto p‐MoTe_2_, followed by 60‐nm‐thick Au capping layers. The work functions of the Cr and Ni metals in this case are lower as compared to those of Pd (*Φ*_Cr_ (4.5 eV) < *Φ*_Ni_ (5.0 eV) < *Φ*_Pd_ (5.3 eV)).^[^ ^27^ ^]^ The inset of Figure 1b shows an optical image of completed FETs with a p‐MoTe_2_ flake, with the thickness determined to be 13 nm (see Figure S1a, Supporting Information).

From MoTe_2_ FETs, we first investigated the device performance in the dark (see Figures S1–S3, Supporting Information). Observed characteristics of the devices include the p‐type nature of MoTe_2_ with a low threshold voltage of ≈8 V and a high ON/OFF current ratio (*I*_on_/*I*_off_) up to 10^4^ (Figure S1b, Supporting Information), as well as high hole mobility (*μ*_FE_)^[^ ^28^ ^]^ of 120 cm^2^ (Vs)^−1^ with the Pd electrodes. Such high hole mobility and the low threshold voltage of the p‐MoTe_2_ FET with the Pd electrodes, as compared to those of other TMD‐based FETs on SiO_2_ substrates,^[^ ^9a^ ^]^ may be due to the high work function of the Pd metal, possibly resulting in a low Schottky barrier height (*φ*_B_≈ 28 meV)^[^ ^29^ ^]^ at the Pd‐MoTe_2_ junction (Figure S2, Supporting Information). Moreover, the linear behaviors of the current–voltage (*I*_ds_–*V*_ds_) curves of the p‐MoTe_2_ FET with Pd verify the Ohmic contact (Figure S3, Supporting Information). The observed key parameters of the p‐MoTe_2_ FETs are summarized in **Table** **1**.

**Table 1:** Comparison of the key parameters of p‐MoTe_2_ and n‐PdSe_2_ FETs and p‐MoTe_2_/n‐PdSe_2_ vdWH FETs

Structure	Electrode	*μ*_FE_ [cm^2^ V^−1^ s−^1^]	*φ*_B_ [meV]	*τ*_r_/*τ*_d_ [ms]	*R* [A W^−1^]	D* [Jones]	*EQE* [%]
p‐MoTe_2_ FET	Pd	120	28	0.5/0.9	5.3 × 10^2^	4.2 × 10^9^	1.6 × 10^3^
	Ni	98	45	3/6	2.9 × 10^2^	1.5 × 10^9^	9.0 × 10^2^
	Cr	56	90	9/11	1.5 × 10^2^	9.2 × 10^8^	4.6 × 10^2^
n‐PdSe_2_ FET	Sc	162	16	0.09/0.07	1.2 × 10^3^	2.1 × 10^11^	3.6 × 10^3^
	Al	102	38	0.1/0.3	3.7 × 10^2^	6.4 × 10^10^	1.1 × 10^3^
	Ti	98	75	0.8/0.9	2.4 × 10^2^	9.5 × 10^9^	7.5 × 10^2^
MoTe_2_/PdSe_2_ vdWH FET	Pd and Sc	–	–	0.01/0.03	1.2 × 10^5^	2.4 × 10^14^	3.5 × 10^6^

Subsequently, optoelectrical measurements of p‐MoTe_2_ FETs were taken under incident laser light with different wavelengths (*λ*s) in the VNIR region for the three different metal electrodes (Pd, Ni, and Cr). Figure 1b shows the *I*_ds_–*V*_ds_ curves of the MoTe_2_ FET with Pd electrodes under incident laser light with different wavelengths in the VNIR region. The drain current *I*_ds_ was increased from 10 µA in the dark to 31 µA at the bias voltage *V*_ds_ of 2.0 V under the illumination of incident visible laser light (*λ* = 532 nm) with an input power of 20 nW. In contrast, under the illumination of incident NIR laser light (*λ* = 1310 nm), the increase in the *I*_ds_ response is quite small due to the relatively large intrinsic bandgap (≈1.1 eV) of MoTe_2_. From these *I*_ds_ data, the photoresponsivity *R*, a pivotal parameter of photodetectors, was also estimated using the relationship *R*  = *I*_ph_ /*PA*,^[^ ^30^ ^]^ where *A*, *I*_ph_, and *P* are the junction area, the photocurrent current ( *I*_ph_ = *I*_ds(light)_  − *I*_ds(dark)_), and the input power of incident light, respectively. The estimated maximum value of *R* for the MoTe_2_ FETs with Pd is 532 A W^−1^ in the visible region (*λ* = 405 nm) at zero back gate voltage (*V*_bg_ = 0 V) (Figure 1c). As expected, in the NIR region, however, the responses of the devices are very close to zero, as shown in the figure. Further detailed descriptions of the electrode effect on *R* and other characteristics of the MoTe_2_ FETs with Pd are described in Figure S4a–c, Supporting Information.

Next, we took electrical measurements of n‐type PdSe_2_ FETs with various metal electrodes in the dark and under incident light. Figure 1d shows a schematic illustration of an n‐PdSe_2_ FET. To characterize the optoelectronic properties of PdSe_2_, we designed n‐PdSe_2_ FETs with different metal electrodes with Sc, Al, and Ti. Here, the Ti and Al metals have higher work functions as compared to those of Sc (*Φ*_Ti_ (4.33 eV) > *Φ*_Al_ (4.3 eV) > *Φ*_Sc_ (3.5 eV)).^[^ ^27^, ^31^ ^]^ The inset of Figure 1e presents an optical image of the completed PdSe_2_ FETs. The thickness of the PdSe_2_ flake is 10 nm (Figure S5a, Supporting Information). From the completed PdSe_2_ FETs, we also investigated the characteristics of the devices in the dark (see Figures S5–S7, Supporting Information). The observed device performance shows the n‐type nature of PdSe_2_ with a high *I*_on_/*I*_off_ up to 10^6^ (Figure S5b, Supporting Information) and high electron mobility *μ*_FE_ of 162 cm^2^ (Vs)^−1^ with the Sc electrodes. Such a high hole mobility of the n‐PdSe_2_ FET with the Sc electrodes may be due to the low work function of the Sc metal, possibly resulting in a low *φ*_B_ of 16 meV at the Sc‐PdSe_2_ junction (Figure S6, Supporting Information). The linear behaviors of the current–voltage (*I*_ds_–*V*_ds_) curves of the n‐PdSe_2_ FET with Sc also verify that the Sc electrode forms a low and negligible Schottky barrier height with Ohmic contact at the TMD‐metal junction (n‐PdSe_2_/Sc) (Figure S7, Supporting Information). The observed key parameters of the n‐PdSe_2_ FETs are also summarized in HYPERLINK ∖l "tb1" Table 1.

Figure 1e presents the optoelectrical characteristics of *I*_ds_–*V*_ds_ for the PdSe_2_ FET with Sc under incident light illumination with different wavelengths (*λ*s) in the VNIR region. Under incident light (*λ* = 532 nm) with an input power of 20 nW, the drain current *I*_ds_ was increased from 3.5 µA in the dark to 60 µA at a bias voltage *V*_ds_ of 2.0 V. Given these observations, we also estimated the *R* values of the PdSe_2_ FETs as a function of the wavelength of incident light. The estimated maximum value of *R* was found to be in the range of 1.2 × 10^3^ A W^−1^, as shown in Figure 1f. Such high *R* values of PdSe_2_ may be caused by the photo‐doping and/or photo‐gating effect.^[^ ^16^, ^32^ ^]^ To confirm this effect, the transfer curves of the FET with Sc were measured under incident light with different *λ*s and compared to those measured in the dark (Figure S8a, Supporting Information), exhibiting illumination‐induced shifts towards positive gate voltages. Such shifts are a clear confirmation of the photo‐gating effect via the increased number of trapping holes at the interface, resulting in additional electron flows in PdSe_2_ under light illumination.^[^ ^16^, ^32^ ^]^ Detailed descriptions of the electrode effect on *R* and other characteristics of the n‐PdSe_2_ FETs are described in Figure S8b–d, Supporting Information.

**Figure 2:**
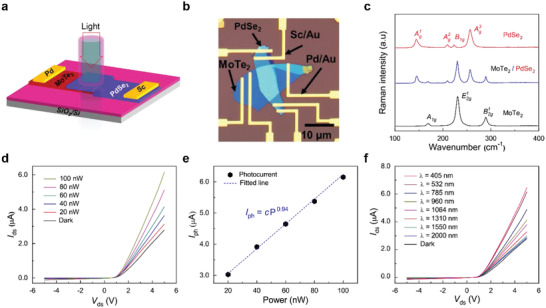
a) Schematic illustration of a vdWH FET with MoTe_2_/PdSe_2_ under incident light illumination. b) Optical image of a MoTe_2_/PdSe_2_ vdWH FET with Pd and Sc electrodes. c) Raman spectra of individual flakes of MoTe_2_ and PdSe_2_ and the MoTe_2_/PdSe_2_ vdWHs. d) *I*_ds_–*V*_ds_ curves of the MoTe_2_/PdSe_2_ vdWH FET with Pd and Sc electrodes under incident laser light (*λ* = 532 nm) with various input power levels. e) Input power dependence of the photocurrent *I*_Ph_ of the MoTe_2_/PdSe_2_ FET for incident light (*λ* = 532 nm) (black dots). The blue dotted line shows the best‐fitted result. f) *I*_ds_–*V*_ds_ curves of the MoTe_2_/PdSe_2_ FET in the dark and under incident light with several different wavelengths in the VNIR region at a given input power of 100 nW.

At this point, we focus our attention on the vdWHs of MoTe_2_ and PdSe_2_ (MoTe_2_/PdSe_2_). We fabricated an FET with MoTe_2_(10 nm)/PdSe_2_(13 nm) vdWHs and then measured its electrical characteristics in the dark and under incident laser light. **Figure** **2**a shows a schematic illustration of the MoTe_2_/PdSe_2_ vdWH FET. Figure 2b shows an optical image of a completed MoTe_2_/PdSe_2_ vdWH FET with Pd and Sc electrodes for the optimization of the Ohmic contact for the vdWHs. In order to investigate the structural fingerprints of p‐MoTe_2_ and n‐PdSe_2_ in the fabricated vdWHs, the Raman spectra of each TMD material and the vertically stacked materials with the heterostructure were measured, as presented in Figure 2c. From the PdSe_2_ flake of the FET, we observed the first three Raman peaks at low wavenumbers ($A_{g}^{1}\approx 144$ cm^−1^, $A_{g}^{2}\approx \;209.2\;$cm^−1^, and *B*_1*g*_ ≈ 223.3 cm^−1^) and the fourth peak at a high wavenumber ($A_{g}^{3}\approx 256.5$ cm^−1^) (upper curve). These peaks correspond to the vibrational modes of Se atoms and the relative vibration mode between the Pd atom and Se atoms.^[^ ^33^ ^]^ For the p‐MoTe_2_ flake, three Raman peaks were observed at A_1g_ ≈  173.6 cm^−1^, $E_{2g}^{1}\approx \;$235.1 cm^−1^, and $\;B_{2g}^{1}\approx \;$290.9 cm^−1^, which belong to the out‐of‐plane, in‐plane, and bulk‐inactive modes, respectively (lower curve). The main Raman peak $E_{2g}^{1}$ of MoTe_2_ is related to the in‐plane vibrations between the Mo and Te atoms.^[^ ^8b^ ^]^ Further, in the MoTe_2_/PdSe_2_ vdW heterostructure, the observed Raman spectra clearly confirm that the basic structural characteristics of both TMD materials are clearly conserved, as shown in the figure (middle curve).

Subsequently, we investigated the device performance of the MoTe_2_/PdSe_2_ vdWH FET. In the dark, we found and verified excellent electric characteristics of the heterojunction device (see Figure S9, Supporting Information). Examples include an efficient gate‐voltage‐controlled effect of the rectification ratio *RR* with a corresponding maximum value of ≈0.63 × 10^6^ due to the considerable suppression of the reverse/leakage current (Figure S9a,b, Supporting Information) via the electric switching characteristic of the TMDs between the semiconducting and semi‐insulating phases,^[^ ^34^ ^]^ and an efficient gate‐voltage‐modulated ideality factor (*η*) with a lower value of 1.1 (Figure S9c,d, Supporting Information), among others. It should be noted that the maximum *RR* value of the MoTe_2_/PdSe_2_ vdWH FET in this study is significantly high in comparison with those of the various TMD devices reported previously (Table S1, Supporting Information).^[^ ^35^ ^]^ Thus, it is clear that effective electrostatic control of the charge transport in MoTe_2_/PdSe_2_ vdWHs can provide a way to design various TMD‐based electronic devices. In addition, it should be noted that when the layer thickness of MoTe_2_ or PdSe_2_ decreased, the device performance began to deteriorate (Figure S10, Supporting Information).

Next, we explored the optoelectric properties of the MoTe_2_/PdSe_2_ vdWH FETs. Figure 2d shows the *I*_ds_ −  *V*_ds_ characteristics as measured under the illumination of incident light (*λ* = 532 nm) with various input powers at *V*_bg_ = 0 V. From the *I*_ds_ −  *V*_ds_ curves, it was found that the forward and reverse photocurrents *I*_Ph_ increase continuously as the input power of the incident laser light increases. Such increases in the reverse and forward photocurrents of the MoTe_2_/PdSe_2_ vdW heterojunction device are mainly due to the efficient generation of photo‐excited electron–hole (*e*–*h*) pairs and the effective separation of excess *e*–*h* pairs caused by the built‐in electric field at the interface of the heterojunction. Figure 2e shows the photocurrent *I*_Ph_ as a function of the input power of incident light (*λ* = 532 nm). In this figure, the photocurrent shows sub‐linear dependence on the input power of incident light, expressed as *I*_Ph_ =  *cP* ^*θ*^, where *c* is a proportional constant, *P* is the input power of the incident light, and *θ* denotes the power‐law index.^[^ ^36^ ^]^ From the best linear fit to the experimental data, the obtained value of the power‐law index *θ* is 0.94, which is very close to 1.0 for an ideal photodetector having a low trap state junction.^[^ ^36^ ^]^ Thus, this result clearly indicates that the MoTe_2_/PdSe_2_ vdWHs have a small number of trap states at the sharp heterojunction interface, which is crucial when attempting to realize highly sensitive photodetectors.

In addition, we studied the dependence of the photoresponse of the MoTe_2_/PdSe_2_ vdWH FET on the wavelength *λ* of incident light in the VNIR range. Figure 2f shows the *I*_ds_ −  *V*_ds_ curves of the MoTe_2_/PdSe_2_ vdWH FET for several different *λ* values of incident light. Interestingly, as indicated in the figure, the current *I*_ds_ of the heterojunction FET decreases slightly as *λ* of the incident light increases. For example, the observed values of *I*_ds_ were 6.45 and 2.85 µA for *λ* = 405 and 2000 nm, respectively. Note that in contrast to the significant decrement of *I*_ds_ of the single TMD‐based FETs with MoTe_2_ or PdSe_2_ in the NIR region (see Figures 1b and 1e), *I*_ds_ of the MoTe_2_/PdSe_2_ vdWH FET decreased slightly as *λ* increased. Such a small decrement of *I*_ds_ of the vdWH FET in the NIR region cannot be explained solely in terms of the intrinsic optical absorption of each TMD material used (MoTe_2_ and PdSe_2_), as discussed below.

**Figure 3:**
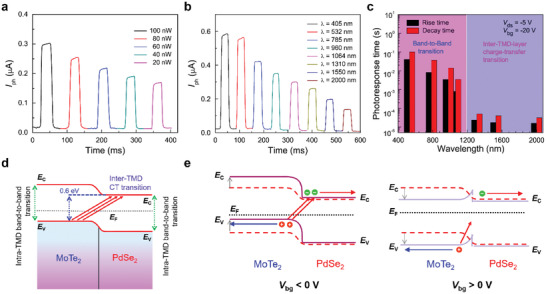
Temporal photocurrents of the MoTe_2_/PdSe_2_ vdWH FET as a function of time under incident light a) with several different input power levels at a fixed *λ* (1064 nm) and b) with several different *λ*s in the VNIR region (*λ* = 405–2000 nm) at a fixed *P* (100 nW) while turning the incident light on and off. c) Rise (*τ*_r_) and decay (*τ*_d_) times of the MoTe_2_/PdSe_2_ vdWH FET at several *λ*s of incident light with *V*_ds_ equal to −5 V and *V*_bg_ set to −20 V. d) Energy band diagram of the MoTe_2_/PdSe_2_ vdWH FET under incident light illumination. e) Energy band diagrams of the MoTe_2_/PdSe_2_ vdWH FET under negative (*V*_bg_ < 0 V, left panel) and positive (*V*_bg_ > 0 V, right panel) back gate voltages. *E*_C_: conduction band, *E*_V_: valence band, and *E*_F_: Fermi level.

Next, we evaluated the temporal photoresponse characteristics of the MoTe_2_/PdSe_2_ vdWH FET while turning the incident light on and off. The temporal photoresponses of the device were measured as a function of time for various input powers (*P*s) of incident light (*λ* = 1064 nm) (**Figure** **3**a) and for various *λ*s of incident light in the VNIR range at a fixed *P* of 100 nW (Figure 3b). From these responses, we estimated the response times, that is, the rise (*τ*_r_) and decay (*τ*_d_) times, using the simple relationship of *I*_tot_ = *I*_dark_  + *c*exp [ ± *t*/*τ*_r,d_], where *I*_tot_ is the temporal photocurrent, *I*_dark_ is the dark current, *c* is a proportional constant, and *t* is the time. By fitting the data, we attained remarkably fast rise and decay times of the vdWH FET, that is, *τ*_r_ = 16.1 µs and *τ*_d_ = 31.1 µs for NIR incident light with a wavelength *λ* of 2000 nm. The obtained response times of the MoTe_2_/PdSe_2_ vdWH FET for several *λ*s are presented in Figure 3c. In comparison, the temporal photoresponses of single TMD FETs with MoTe_2_ or PdSe_2_ were also measured for several different electrodes (Figure S11, Supporting Information). The estimated *τ*_r_/*τ*_d_ values of the MoTe_2_ FETs were 0.5/0.9, 3.0/6.0, and 9.0/11.0 ms for the Pd, Ni, and Cr electrodes, respectively, while the estimated *τ*_r_/*τ*_d_ values of the PdSe_2_ FETs were 0.1/0.1, 0.1/0.3, and 0.7/0.9 ms for the Sc, Al, and Ti electrodes, respectively. Thus, both the MoTe_2_ and PdSe_2_ FETs exhibited relatively slow rise and decay times, with their operating wavelength ranges restricted to the visible wavelength region due to their limited optical absorption ranges (*λ* <980 nm) via the electronic transition from the valence band to the conduction band, that is, intra‐TMD band‐to‐band transitions across the corresponding intrinsic bandgap Δ*E* ($\Delta E_{\mathrm{MoT}e_{2}}\approx 1.03\;\;\mathrm{eV}\;\mathrm{and}\;\delta E_{\mathrm{PdS}e_{2}}\approx 0.81\;\;\mathrm{eV})$. In contrast, for the MoTe_2_/PdSe_2_ vdWH FET, in addition to the electronic intra‐TMD band‐to‐band transitions across the intrinsic bandgap in this case, another type of electronic transition is possible between MoTe_2_ and PdSe_2_, that is, an inter‐TMD charge‐transfer (CT) transition between the MoTe_2_ and PdSe_2_ TMDs, as illustrated in Figure 3d. This type of optical transition occurs across from the valence band maximum (VBM) of MoTe_2_ to the conduction band minimum (CBM) of PdSe_2_, that is, a MoTe_2_‐to‐PdSe_2_ CT transition. For such an inter‐TMD CT transition, a smaller amount of energy is required ($\Delta E_{\mathrm{MoT}e_{2}-\mathrm{to}-\mathrm{PdS}e_{2}}$) compared to that required for the intrinsic intra‐TMD band‐to‐band optical transition of each individual TMD material. Therefore, $\Delta E_{\mathrm{MoT}e_{2}-\mathrm{to}-\mathrm{PdS}e_{2}}$ is small enough (< 0.6  eV) to be feasible for use in the NIR region (*λ* > 980 nm) up to *λ* = 2000 nm.

Moreover, it is noteworthy that such an inter‐TMD CT transition in the MoTe_2_/PdSe_2_ heterostructure device results in significantly fast photoresponses, especially for the NIR region, as shown in Figure 3c. The remarkably short response times related to the inter‐TMD CT transition in the long‐wavelength region (*λ* > 980 nm) can be ascribed to the quick separation into photo‐carriers and the rapid charge carrier transfer at the interface of the MoTe_2_/PdSe_2_ vdWHs. It should be noted that the observed rise and decay times of the MoTe_2_/PdSe_2_ vdWH FET are one order of magnitude faster than those of other TMD‐based heterostructure devices reported previously.^[^ ^11^, ^23^, ^37^ ^]^

Interestingly, it was also found that the response times of the MoTe_2_/PdSe_2_ vdWH FET studied here are strongly affected by the back gate voltage *V*_bg_. In order to explain this behavior, energy‐level band diagrams of the MoTe_2_/PdSe_2_ FET under negative and positive *V*_bg_ levels are presented in Figure 3e. Under negative back gate voltage (*V*_bg_ < 0 V), the energy level of the bottom MoTe_2_ TMD shifted up, which enhanced the strength of the electric field (*E*) at the interface between the MoTe_2_ and PdSe_2_ layers in the vdWHs, subsequently facilitating the rapid separation and collection of numerous photo‐carriers via inter‐TMD CT transitions. Thus, the photoresponse times, *τ*_r_ and *τ*_d_, decreased significantly to 16.1 and 31.1 µs (at *V*_bg_ = −20 V and *V*_bias_ = −5 V), respectively, even for incident light in the NIR region (*λ* = 2000 nm) (Figures 3c and 3d). In contrast, under positive back gate voltage (*V*_bg_ > 0 V), the energy level of MoTe_2_ moved downward and thus the strength of *E* and the CT transition decreased, resulting in reductions of the separation and collection of photo‐carriers with slow response times (*τ*_r_/*τ*_d_ ≈ 65.6/92.8 µs).

**Figure 4:**
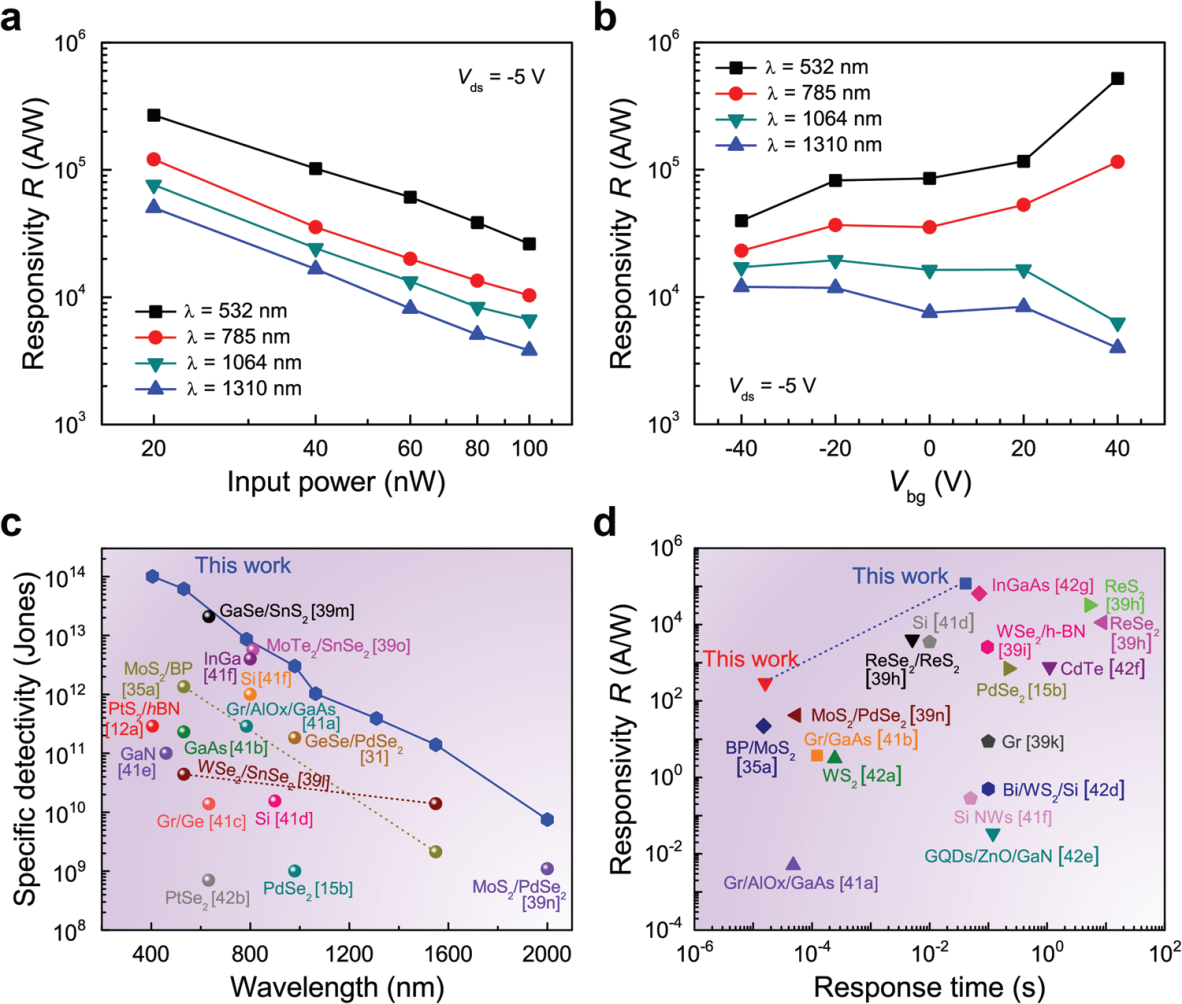
Fundamental parameters of the MoTe_2_/PdSe_2_ vdWH FET: a) photoresponsivity (*R*) of the MoTe_2_/PdSe_2_ FET as a function of the input power *P* of incident light with various *λ*s at *V*_ds_ = −5 V. b) *R* as a function of *V*_bg_ for incident light with several different *λ*s. c) Specific detectivity D* of the MoTe_2_/PdSe_2_ vdWH FET with Pd and Sc contact electrodes as a function of *λ* together with comparisons of those of several TMD photo‐detecting devices reported previously. d) Several photodetector technologies plotted against their corresponding responsivity levels and time responses. The top left region corresponds to high‐gain‐bandwidth‐product detectors and would be the ultimate performance target of future photodetectors.

Next, we analyzed the characteristic parameters, in this case the photo‐switching ratio (*I*_ph_/*I*_dark_) and the responsivity *R* of the MoTe_2_/PdSe_2_ vdWH FET. From the data shown in Figure 3a,b, we estimated the photo‐switching ratio *I*_ph_/*I*_dark_ of the vdWH FET and found that the *I*_ph_/*I*_dark_ ratio is extremely high, reaching nearly ≈  10^4^ mainly due to the enhanced photocurrent *I*_ph_ as well as the suppressed dark current *I*_dark_ in the vdWH FET (Figure S12, Supporting Information).^[^ ^30a^ ^]^ The responsivity *R* values of the vdWH FET were then extracted as a function of the input power of incident light with several different wavelengths (**Figure** **4**a). As shown in the figure, high *R* values were obtained due to the small bandgap with low Schottky contact barriers in the VNIR region. The responsivity *R* is significantly higher over the broad spectral range due to the intensification and separation of photo‐carriers in the vdWH FET device^[^ ^38^ ^]^ via the intra‐TMD band‐to‐band transitions together with the inter‐TMD CT transitions under the internal electric field at the interface between the PdSe_2_ and MoTe_2_, as mentioned above. Thus, a high value of *R* can be achieved in the MoTe_2_/PdSe_2_ vdWH FET over a broad VNIR spectral range.

The responsivity *R* values of the MoTe_2_/PdSe_2_ vdWH FET were also observed as a function of *V*_bg_ for several wavelengths of incident light in the VNIR range (Figure 4b). For incident light at a short wavelength ****λ**** (visible range), as the gate voltages decrease (*V*_bg_ < 0), the *R* value also decreases continuously. This is caused by the reduced collection of photo‐carriers generated via the intra‐TMD band‐to‐band transitions at a negative *V*_bg_ owing to the increased effective *φ*_B_ at the interface between the MoTe_2_ TMD and Pd electrode. In contrast, for incident light with a long wavelength ****λ**** (NIR range), the value of *R* increases with a decrease in *V*_bg_. In this NIR range, a large amount of photocurrent can be generated due to the inter‐TMD CT transition between MoTe_2_ and PdSe_2_ at *V*_bg_ < 0. It is therefore clear that not only the intra‐TMD band‐to‐band transition but also the inter‐TMD CT transition for the MoTe_2_/PdSe_2_ vdWH FET can be efficiently controlled by the back gate voltage, leading to a high value of *R* (≈>10^4^–10^6^) in the broad VNIR region. We also investigated the effects of the metal contacts on the *R* values. As shown in Figure S13a, Supporting Information, the Ohmic contact with Pd and Sc electrodes for the MoTe_2_/PdSe_2_ vdWHs (Pd/MoTe_2_/PdSe_2_/Sc) show the best results among the metal contacts studied here, mainly due to the small values of *φ*_B_ for the Pd and Sc metal contacts. The maximum value of *R* for the contact‐optimized MoTe_2_/PdSe_2_ vdWH FET reached 1.2 × 10^5^ A W^−1^, which is significantly higher than previously reported values^[^ ^15^, ^30^, ^39^ ^]^ (Figure S13b, Supporting Information).

Furthermore, the specific detectivity (D*) of MoTe_2_/PdSe_2_ vdWH FETs with Pd and Sc contact electrodes were also estimated using the relationship $\;D^{*}=\;\sqrt{A\Delta f}/\sqrt{\left\langle i_{n}^{2}\right\rangle }\;R$,^[^ ^12^, ^39^, ^40^ ^]^ where Δ*f* and *i*_n_ represent the bandwidth and noise current, respectively. The mean square noise current $\left\langle i_{n}^{2}\right\rangle$ is calculated according to $\left(\;\left\langle i_{n}^{2}\right\rangle =\;4k_{B}T\Delta f/R_{0}\right)$, where Δ*f* and *R*_0_ correspondingly denote the bandwidth and device resistance. The estimated mean square noise current is 1.8 ×10^−25^ A^2^ Hz^−1^. A remarkably high value of D* of 2.4 × 10^14^ Jones is achieved, which is nearly two orders of magnitude higher than those previously reported,^[^ ^12^, ^39^, ^40^, ^41^ ^]^ as shown in Figure 4c. Even at room temperature, the MoTe_2_/PdSe_2_ vdWH FET exhibits a significantly wide spectral response from 400 to 2000 nm, with D* greater than 10^14^–10^12^ Jones at wavelengths from 400 to 1064 nm, greater than 10^11^ Jones from 1064 to 1550 nm, and greater than 10^9^ Jones from 1550 to 2000 nm. Such high detectivity with a broad response of the MoTe_2_/PdSe_2_ vdWH FET is comparable to or even better than those from conventional Si and GaAs photodetectors.^[^ ^41b,e,f^ ^]^ Moreover, the *EQE*s of the MoTe_2_/PdSe_2_ vdWH FET were also extracted using the relationship $EQE\;=\;R\frac{hc}{q\lambda }\;$(Figure S13c, Supporting Information). As shown in the figure, the obtained *EQE* values were considerably high, reaching *EQE* ≈3.5 ×10^6^ at *λ* = 405 nm and *EQE* ≈1.3 ×10^2^ at *λ* = 2000 nm, much greater as compared to previous values, as shown in the figure, especially for the NIR region.^[^ ^15^, ^39^, ^42^ ^]^ In addition, the long‐term storage stability of the MoTe2/PdSe_2_ vdWH FET was also studied, as presented in Figure S14, Supporting Information.

Finally, in Figure 4d, we show the responsivity outcomes and response times of several important photodetector technologies, including the MoTe_2_/PdSe_2_ vdWH FET studied here. It is clear from the figure that the response time of the MoTe_2_/PdSe_2_ vdWH FET is considerably faster as compared to previous values, especially for the VNIR region, without sacrificing other key figures of merit such as the responsivity and *EQE*. Thus, the MoTe_2_/PdSe_2_ vdWHs studied here hold great promise for fast and high photo‐detectivity over a wide spectral range in the VNIR region. Such significantly improved values of *R*, D*, and *EQE* in the broad VNIR region with fast response times mainly stem from the excellent Ohmic contact with low Schottky barriers, the sharp interface, and the inter‐TMD CT transition with intra‐TMD band‐to‐band transitions in the MoTe_2_/PdSe_2_ vdWHs. Hence, the MoTe_2_/PdSe_2_ vdWHs studied here can be a useful for improving the photocurrent and photo‐gating characteristics, with possible applications in various optoelectronics in the VNIR region.

Overall, the findings above show that the photoresponse characteristics of FETs with the TMD heterostructure, such as MoTe_2_/PdSe_2_ vdWHs, are mainly influenced by the metal‐TMD contacts and the CT transition between MoTe_2_/PdSe_2_ vdWHs, which will provide a key platform for further improvements in the performance of VNIR photodetectors coupled with TMD materials.

## 3 Conclusion

In summary, we reported a novel TMD heterostructure assembly consisting of MoTe_2_ and PdSe_2_. Effective tuning of the rectification ratio *RR* of 6.3 × 10^5^ at *V*_bg_ = −40 V with a low *φ*_B_ value and sharp interface is realized for the MoTe_2_/PdSe_2_ vdWH FET with Pd and Sc electrodes. This *RR* value is much higher than those of other TMD vdWHs reported previously. Excellent photoresponse performance capabilities of the MoTe_2_/PdSe_2_ vdWH FET were noted in the VNIR region (*λ*   =   405  −  2000  nm). Rapid rise (16.1 µs) and decay (31.1 µs) times were also obtained with NIR light (*λ* = 2000 nm). Moreover, extraordinary *R* = 1.2 × 10^5^ A W^−1^, D* (=2.4 × 10^14^ Jones), and *EQE* (=3.5 × 10^6^) outcomes were obtained in the MoTe_2_/PdSe_2_ vdWH FET. These significantly improved values of *R*, D*, and *EQE* mainly stem from the excellent Ohmic contact with low Schottky barriers, the sharp interface, and the inter‐TMD charge‐transfer transition together with intra‐TMD band‐to‐band transitions in the MoTe_2_/PdSe_2_ vdWHs. Therefore, the TMD‐based vdWHs of MoTe_2_/PdSe_2_ studied here represent a new opportunity to develop state‐of‐the‐art photonic devices in optoelectronics, such as effective VNIR optical sensors, waveguide‐integrated photodetectors, and/or nano‐photodetectors.

## 4 Experimental Section

A multilayer MoTe_2_ or PdSe_2_ nanoflake was exfoliated and transferred onto a p‐type Si/SiO_2_ (300 nm) substrate, acting as a back gate, with the help of the standard mechanical exfoliation method using Scotch tape.^[^ ^17a^ ^]^ The thickness of the transferred flake (MoTe_2_ or PdSe_2_) on the substrates was identified by the interference effect.^[^ ^43^ ^]^ Further, Raman spectroscopy and atomic force microscopy were utilized to confirm the thicknesses of the flakes. Only those flakes that had a uniform thickness and clean surfaces were used for the electric and optoelectric measurements.

In order to fabricate the MoTe_2_/PdSe_2_ vdWHs, a multilayer PdSe_2_ nanoflake but also exfoliated and transferred onto the top of a polydimethylsiloxane (PDMS) stamp by the mechanical exfoliation method. Next, with a micro‐aligner stage, the nanoflake with multilayer PdSe_2_ on the PDMS stamp was transferred onto a multilayer MoTe_2_ flake prepared on a Si/SiO_2_ substrate. Subsequently, the MoTe_2_/PdSe_2_ vdWHs were placed in a furnace for thermal annealing at 200 °C under an Ar/H_2_ (97.5%/2.5%) gas flow. After annealing, metal electrodes on the flakes were formed and patterned by electron‐beam deposition and lithography with poly(methyl methacrylate) (PMMA). For the metal electrodes, thin layers (=6 nm) of different metals having high work functions (Pd, Ni, and Cr for MoTe_2_) or low work functions (Sc, Al, and Ti for PdSe_2_) were deposited. This step was followed by the subsequent deposition of 60‐nm‐thick Au capping layers by electron‐beam evaporation. After the deposition of the metal electrodes, the samples were placed in acetone for the lift‐off process. The final sample FET devices were then placed into a vacuum box to measure their electrical performance capabilities.

The fabricated TMD FETs were analyzed according to their transfer curves (*I*_ds_–*V*_bg_) at a constant *V*_ds_ = 1.0 V using a Keithley source voltmeter (K‐2400) and a picometer (K‐6485). The back‐gate voltage (*V*_bg_) was swept between *V*_bg_ = ±40 V and the drain current (*I*_ds_) was determined using the picometer. For optical characterization, the sample FET and heterostructure devices were illuminated under incident laser light with different input powers and wavelengths.

## Conflict of Interest

The authors declare no conflict of interest.

## Supporting information

Supporting Information

## Data Availability

Research data are not shared.
