# Supplementary material for: Highly Sensitive, Ultrafast, and Broadband Photo‐Detecting Field‐Effect Transistor with Transition‐Metal Dichalcogenide van der Waals Heterostructures of MoTe2 and PdSe2
Source: Adv Sci (Weinh). 2021 Mar 16;8(11):2003713. doi: 10.1002/advs.202003713 (PMC8188193; doi:10.1002/advs.202003713)
Supplement: Supplementary file 1 — Supporting Information [file ADVS-8-2003713-s001.pdf]

WILEY-VCH

© 2021 Wiley-VCH GmbH

ADVANCED SCIENCE
Open Access

Supporting Information

for *Adv. Sci.,* DOI: 10.1002/advs.202003713

Highly Sensitive, Ultrafast, and Broadband Photo-detecting Field-effect Transistor with Transition-metal Dichalcogenide van der Waals Heterostructures of $MoTe_2$ and $PdSe_2$

Amir Muhammad Afzal, Muhammad Zahir Iqbal, Ghulam Dastgeer, Aqrab ul Ahmad,

*Byoungchoo Park**

Supporting Information

Highly sensitive, ultrafast, and broadband photo-detecting field-effect transistor with transition-metal dichalcogenide van der Waals heterostructures of $MoTe_2$ and $PdSe_2$

Amir Muhammad Afzal, Muhammad Zahir Iqbal, Ghulam Dastgeer, Aqrab ul Ahmad,

*Byoungchoo Park**

Dr. A.M. Afzal, Prof. Dr. B. Park
Department of Electrical and Biological Physics, Kwangwoon University, Wolgye-Dong, Seoul 01897, South Korea
E-mail: bcpark@kw.ac.kr
Dr. M.Z. Iqbal
Nanotechnology Research Laboratory, Faculty of Engineering Sciences GIK Institute of Engineering Sciences and Technology, Topi, 23640, Khyber Pakhtunkhwa, Pakistan
Dr. G. Dastgeer
School of Physics, Peking University, Beijing 100871, China
IBS Center for Integrated Nanostructure Physics, Sungkyunkwan University, Suwon 16419, South Korea
Dr. A. Ahmad
School of Physics and School of Microelectronics, Dalian University of Technology, Dalian, 116000, China

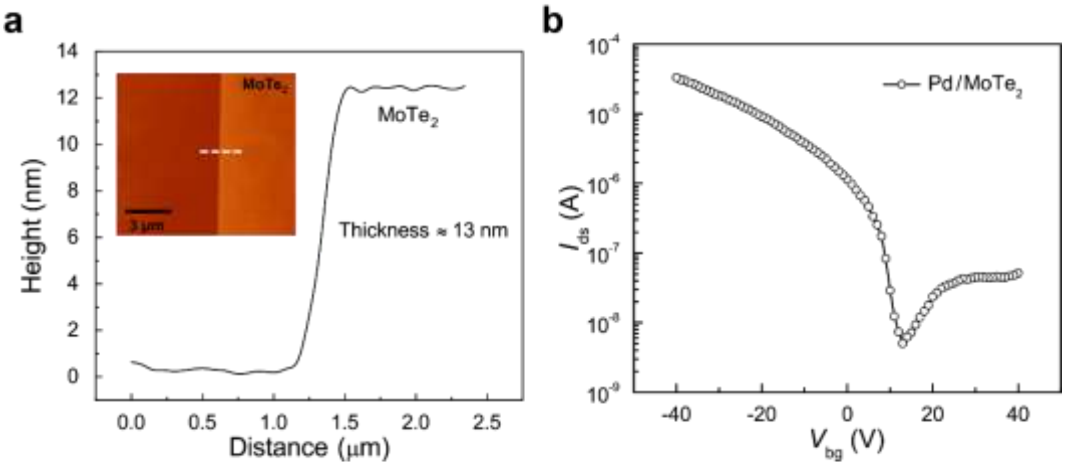

Figure S1. a) Atomic force microscopy (AFM) characterization of a $MoTe_2$ nanoflake with the corresponding height profile. b) A transfer characteristic curve of a p-$MoTe_2$ FET with Pd metal electrodes with the source-drain voltage V_{ds} = 1 V.

Mobilities of the p-$MoTe_2$ FET devices

Figure S1b shows the transfer curve of the p-$MoTe_2$ FET with Pd electrodes at a given source-drain voltage (V_{ds} = 1.0 V) in the dark, clearly confirming the type of the charge carrier (hole) in the p-$MoTe_2$. From the transfer curve of the p-$MoTe_2$ FET, a low threshold voltage was observed at approximately 8 V with a high ON/OFF current ratio (I_{on}/I_{off}) up to 10^4. The charge carrier mobility (μ_{FE}) of the FET was estimated using the relationship [1]

$$\mu_{FE} = \frac{L}{W}\left(\frac{dI_{\mathrm{ds}}}{dV_{\mathrm{bg}}}\right)\frac{1}{C_{\mathrm{bg}}V_{\mathrm{ds}}}, \quad (1)$$

where L, W, and C_{bg} indicate the length, width of the channel, and gate capacitance of the FET, respectively. The estimated hole mobilities μ_{FE}s of the p-$MoTe_2$ FETs with Pd, Ni, and Cr are approximately 120, 98, and 55 cm^2 $(Vs)^{-1}$, respectively.

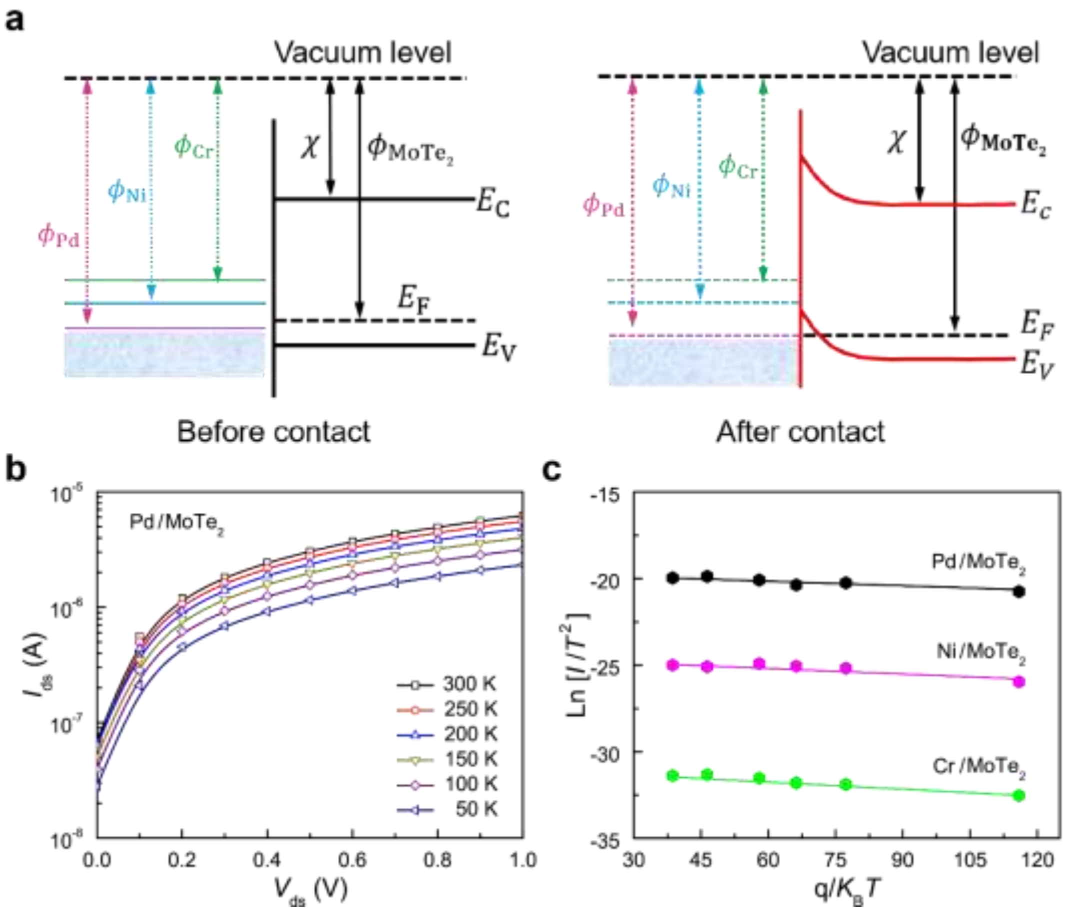

Figure S2. a) Energy band diagrams of three different metal electrodes with $MoTe_2$ before (left) and after (right) contact. Φ_{Pd}, Φ_{Ni}, and Φ_{Cr} represent the work functions of the metal electrodes (Pd, Ni, and Cr). b) I_{ds}- V_{ds} curves of a p-$MoTe_2$ FET at different temperatures on a semi-log scale at zero gate voltage. c) Richardson's plot ($\ln(I/T^2)$ vs q/k_BT) of p-$MoTe_2$ FETs with three different electrodes.

Schottky barrier heights of the p-$MoTe_2$ FET devices

The energy band diagrams of the p-$MoTe_2$ FET devices are shown in **Figure S2a** before and after contact. To investigate the Schottky barrier heights between the metal electrodes (Pd, Ni, and Cr) and the p-$MoTe_2$ TMD material (metal–TMD junction), electrical measurements of the current-voltage (I_{ds}-V_{ds}) characteristics were taken at several different temperatures (*T*s) in the dark, as shown in Figure S2b for the FET with Pd electrodes. The Schottky barrier heights

(ϕ_Bs) between the metal electrodes (Pd, Ni, and Cr) and p-$MoTe_2$ were estimated using the standard thermionic emission model, expressed as [2]

$$I_{ds} = AA^{*}T^{2}\exp\left(\frac{-q\phi_B}{k_B T}\right)\left[\exp\left(\frac{qV}{k_B T}\right) - 1\right], \quad (2)$$

where the symbol A represents the junction area, A^* denotes the Richardson constant, q is the elementary charge, and k_B is the Boltzmann's constant. Based on the above relationship, $\ln(I_{ds}/T^2)$ plots against $(q/k_B T)$ were used to estimate the ϕ_B value at the metal-TMD junctions for the Pd, Ni, and Cr electrodes (Figure S2c). The estimated ϕ_B values are 28, 45, and 90 meV for the Pd, Ni, and Cr electrodes, respectively, as summarized in **Table 1**. Moreover, the linear behaviors of the current-voltage (I_{ds}-V_{ds}) curves of the p-$MoTe_2$ FET with Pd with different values of V_{bg}, as compared to those with other metal contacts of Cr or Ni, verify the Ohmic contact behavior due to the low ϕ_B at the Pd metal-$MoTe_2$ TMD junction (Figure S3). These findings demonstrate that the Pd electrode forms the lowest Schottky barrier height with Ohmic contact at the TMD-metal junction ($MoTe_2$/Pd).

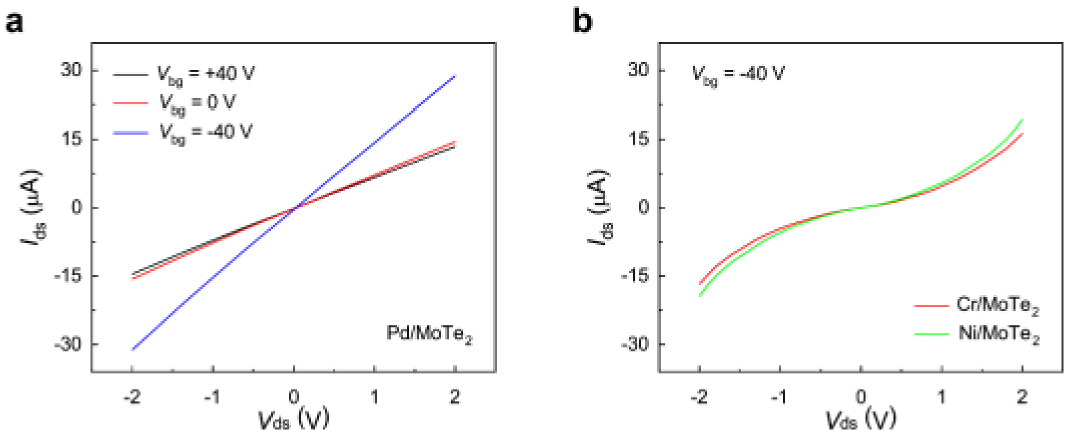

Figure S3. a) Linear $I_{ds} - V_{ds}$ curves of the p-$MoTe_2$ FET with Pd electrodes for several different values of V_{bg}, exhibiting Ohmic contact behavior. b) Non-linear $I_{ds} - V_{ds}$ curves of p-$MoTe_2$ FETs with Ni and Cr electrodes, exhibiting non-Ohmic contact behavior.

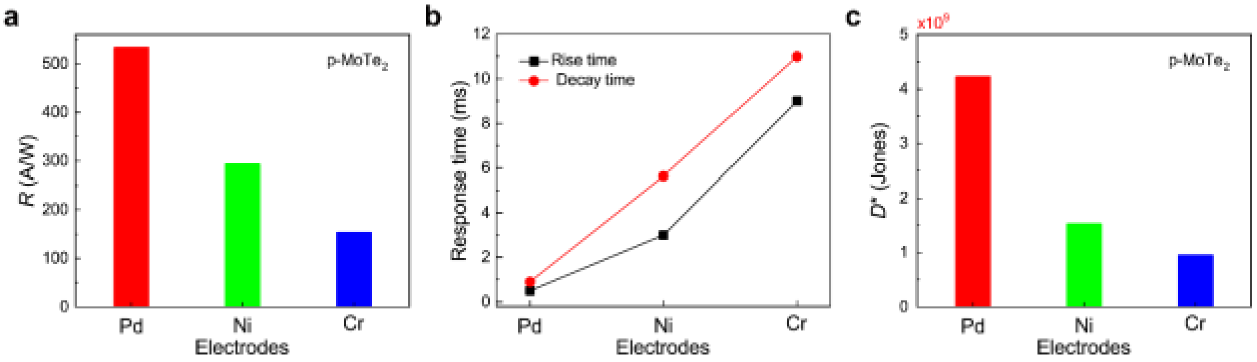

Figure S4. a) Responsivity *R* of p-$MoTe_2$ FETs with respect to three different metal electrodes (Pd, Ni, and Cr) (λ = 405 nm). b) Rise and decay times of the $MoTe_2$ FETs (λ = 405 nm). c) Changes in the specific detectivity (*D**) of the $MoTe_2$ FETs with three different metal electrodes (λ = 405 nm).

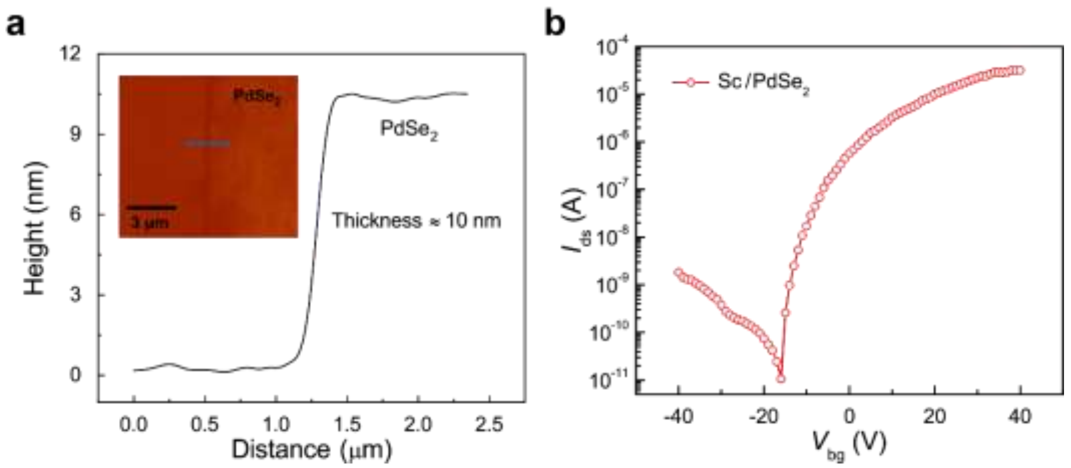

Figure S5. a) AFM image of a $PdSe_2$ nanoflake with the corresponding height profile, and b) a transfer characteristic curve of an n-$PdSe_2$ FET at V_{ds} = 1.0 V.

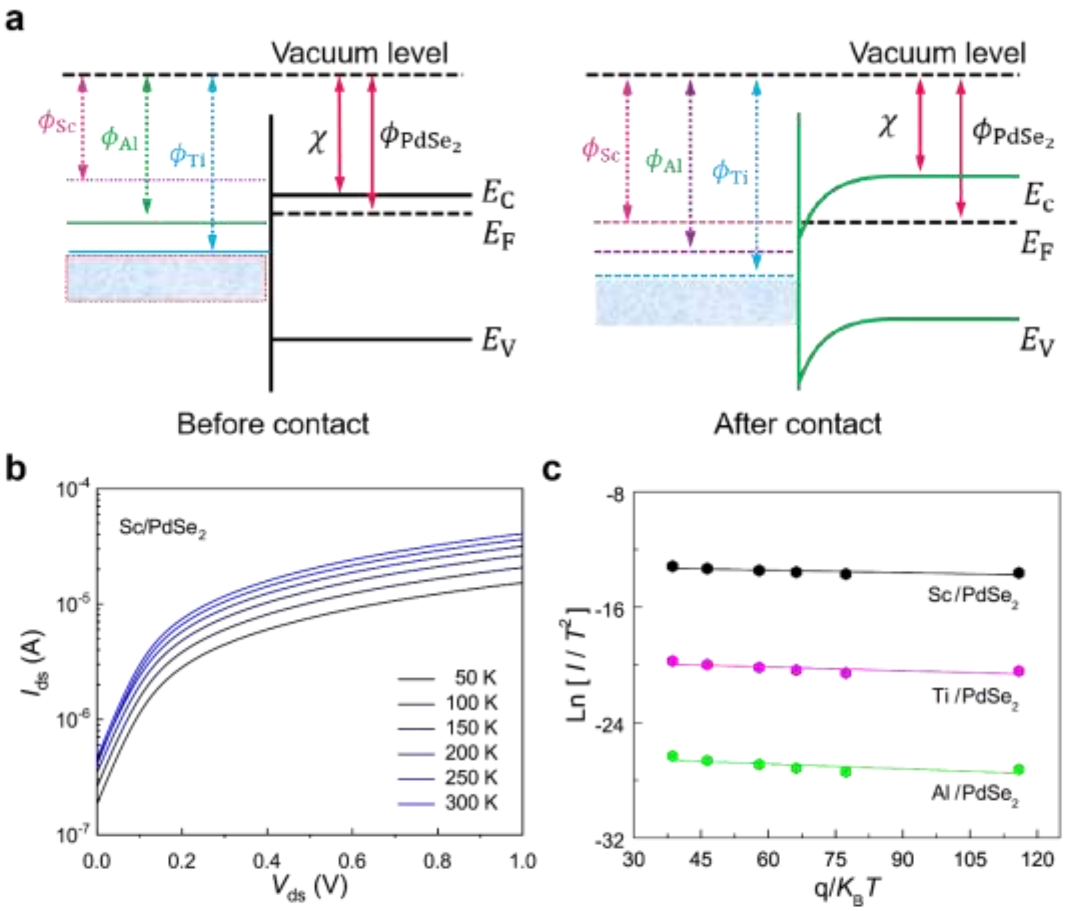

Figure S6. a) Energy band diagrams of three different metal electrodes with $PdSe_2$ before (left) and after (right) contact. $\Phi_{Sc}, \Phi_{Al},$ and Φ_{Ni} represent the work functions of the metal electrodes (Sc, Al, and Ti). b) $I_{ds} - V_{ds}$ curves of the n-$PdSe_2$ FET with Sc electrodes at different temperatures on a semi-log scale at zero back gate voltage. c) Richardson's plot ($\ln(I/T^2)$ vs q/K_BT) of n-$PdSe_2$ FETs with three different electrodes.

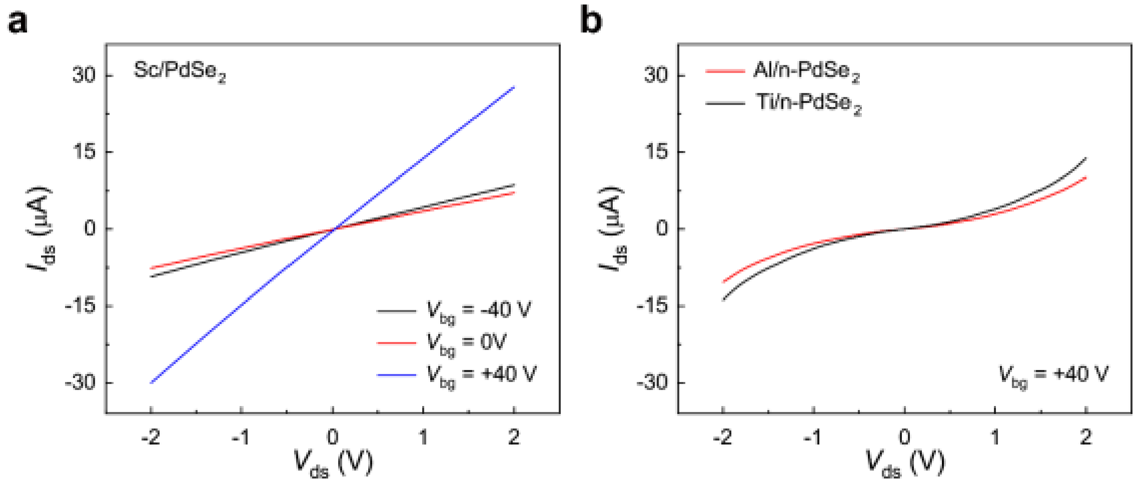

Figure S7. a) Linear $I_{ds} - V_{ds}$ curves of the n-$PdSe_2$ FET with Sc electrodes for several different values of V_{bg}, exhibiting Ohmic contact behavior. b) Non-linear $I_{ds} - V_{ds}$ curves of the n-$PdSe_2$ FETs with Al and Ti electrodes, exhibiting non-Ohmic contact behavior.

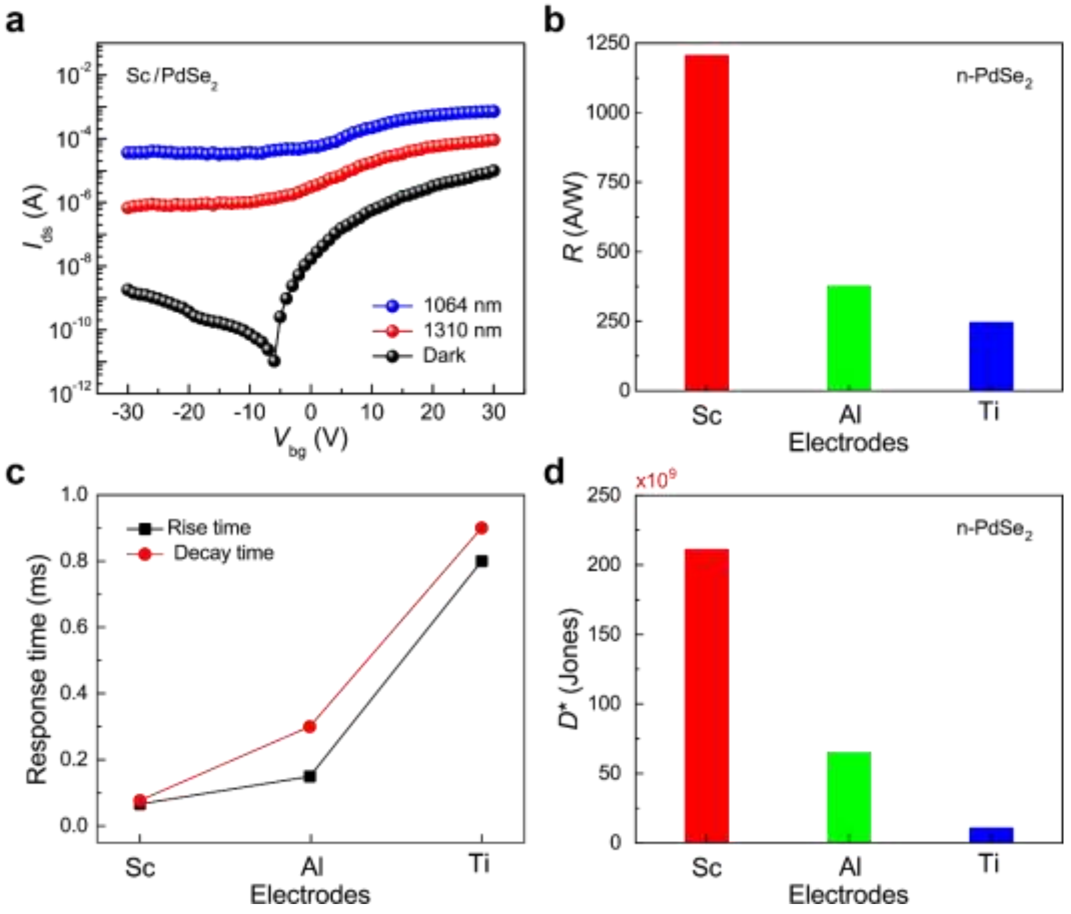

Figure S8. a) Transfer characteristic curves of the $PdSe_2$ FET at V_{ds} = 1.0 V in the dark and under incident light with different wavelengths at a given input power of 100 nW. b)

Responsivity *R* of n-$PdSe_2$ FETs with three different metal electrodes (Sc, Al, and Ti) (λ = 405 nm). c) Rise and decay times of the n-$PdSe_2$ FETs (λ = 405 nm). d) Changes in the specific detectivity (*D**) of the n-$PdSe_2$ FETs with three different metal electrodes (λ = 405 nm)

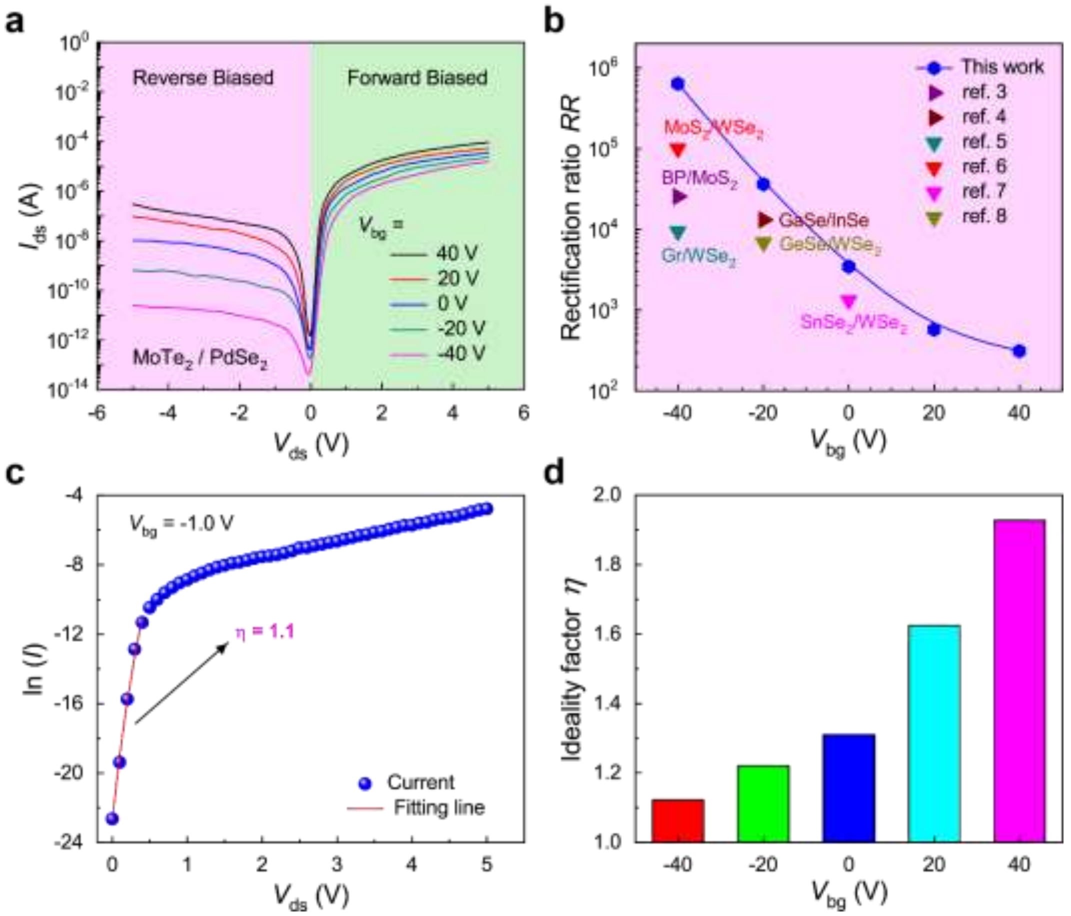

Figure S9. a) Gate voltage (V_{bg})-dependent rectifying current of a $MoTe_2/PdSe_2$ vdWH FET. b) Rectification ratio *RR* of the $MoTe_2/PdSe_2$ vdWH FET as a function of V_{bg} for comparisons with previously reported values.[3] c) Calculation of the ideality factor η and d) η as a function of V_{bg}.

Measurements of the electronic properties of vdWH FETs in the dark

The current-voltage (I_{ds}-V_{ds}) characteristics of the $MoTe_2/PdSe_2$ vdWH FETs at different back-gate voltages (V_{bg}) in the dark are shown in **Figure S9a**. The forward and reverse

currents were measured at the forward and reverse regions, respectively. At V_{ds} < 0 V, the barrier height was increased across the $MoTe_2/PdSe_2$ junction and I_r was decreased. At V_{ds} > 0 V, I_f increased due to the reduction of the barrier height across the $MoTe_2/PdSe_2$ junction. Here, the rectification ratios between the forward current (I_f) measured at V_{ds} = +5.0 V and the reverse current (I_r) measured at V_{ds} = - 5.0 V (rectification ratio (RR) = I_f/I_r) were determined,[3] as shown in Figure S9b. Comparisons of the rectification ratios with previously reported values are also shown in the figure. Moreover, the reverse current through the $PdSe_2$ is low as compared to that with $MoTe_2$, which also causes an increase in the rectification ratio at a negative gate voltage.

The drain current (I_{ds}) of the $MoTe_2/PdSe_2$ FET can be analyzed by the Shockley diode equation, as[4]

$$I = I_o \left[exp\left(\frac{qV}{\eta kT}\right) - 1\right], \tag{3}$$

where I_o is the reverse saturation current, q is the elementary charge, $\eta = \left(\frac{q}{kT}\right)\left(\frac{dV}{dlnI}\right)$ is the ideality factor, k_B denotes the Boltzmann constant ($k_B = 1.3807 \times 10^{-23}$ J/K), and T indicates the absolute temperature. The ideality factor η is determined by finding the slope of the ln $(I_{ds}) - V_{ds}$ curve in the linear forward-bias region at a given gate voltage ($V_{bg} = -40\,V$) (Figure S9c). Figure S9d shows the variation of η as a function of V_{bg}. As shown in the figure, η was determined to be 1.1 at $V_{bg} = -40\,V$, which is very close to the value of η of an ideal device ($\eta = 1.0$). This result provides evidence of the significant improvement in the gate-modulated η for the $MoTe_2/PdSe_2$ FET.[5]

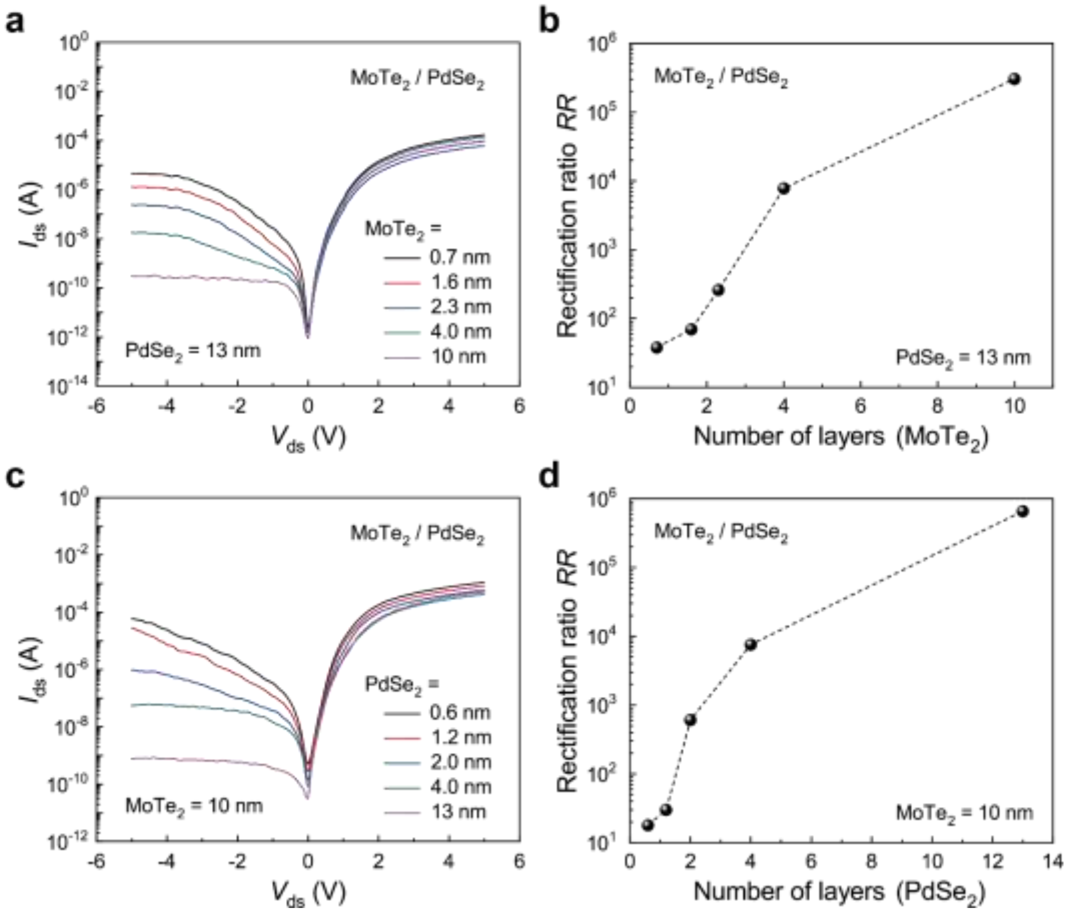

Figure S10. a) I_{ds}-V_{ds} characteristics of $MoTe_2/PdSe_2$ vdWH FETs with several different thicknesses of $MoTe_2$. The thickness of $PdSe_2$ remains constant (13 nm). b) Changes in the rectification ratio with the thickness of $MoTe_2$. c) I_{ds}-V_{ds} characteristics of $MoTe_2/PdSe_2$ vdWH FETs with several different thicknesses of $PdSe_2$. The thickness of $MoTe_2$ remains constant (10 nm). d) Changes in the rectification ratio with the thickness of $PdSe_2$.

Effect of the thickness of the TMD material on the device performance of $MoTe_2/PdSe_2$ vdWH FETs

We studied the effect of the TMD material thickness on the performance of $MoTe_2/PdSe_2$ vdWH FETs. In order to investigate the effect of the thickness, we fabricated and characterized $MoTe_2/PdSe_2$ vdWH FETs with different thicknesses of each TMD material. First, when assessing the $MoTe_2/PdSe_2$ vdWHs, the thickness of $MoTe_2$ was varied from 0.7 nm to 10 nm while the thickness of $PdSe_2$ remained constant (13 nm). The observed I_{ds}-V_{ds}

and the estimated rectification ratio (*RR*) characteristics of $MoTe_2/PdSe_2$ vdWH FETs are shown in **Figures S10a** and S10b, respectively. As shown in these figures, *RR* clearly decreased with a decrease in the thickness of $MoTe_2$. Similarly, for the $MoTe_2/PdSe_2$ vdWHs, the thickness of $PdSe_2$ was varied from 0.6 nm to 13 nm while the thickness of $MoTe_2$ remained constant (10 nm). The observed I_{ds}-V_{ds} and the estimated *RR* characteristics of $MoTe_2/PdSe_2$ vdWH FETs are shown in Figures S10c and S10d, respectively. In this case, the *RR* value also decreased as the thickness of $PdSe_2$ decreased. It is thus noted that the rectifying behavior of the diode is strongly dependent on the thickness of each TMD layer.[6] For the single and bi-layer cases, the barrier width is very narrow and electrons can easily tunnel through the thin depletion region. Thus, ineffective rectification occurs. In contrast, in the multi-layer case, rectifying behavior with a large rectification ratio (> 10^5) was clearly observed. It was also reported that the highest mobility of $PdSe_2$ can be obtained at a thickness of 10-20 nm.[7] Thus, we mainly studied 10-nm-thick $MoTe_2$ and 13-nm-thick $PdSe_2$ flakes in vdWH FETs in an effort to determine the optimum performance for the $MoTe_2/PdSe_2$ vdWH FET.

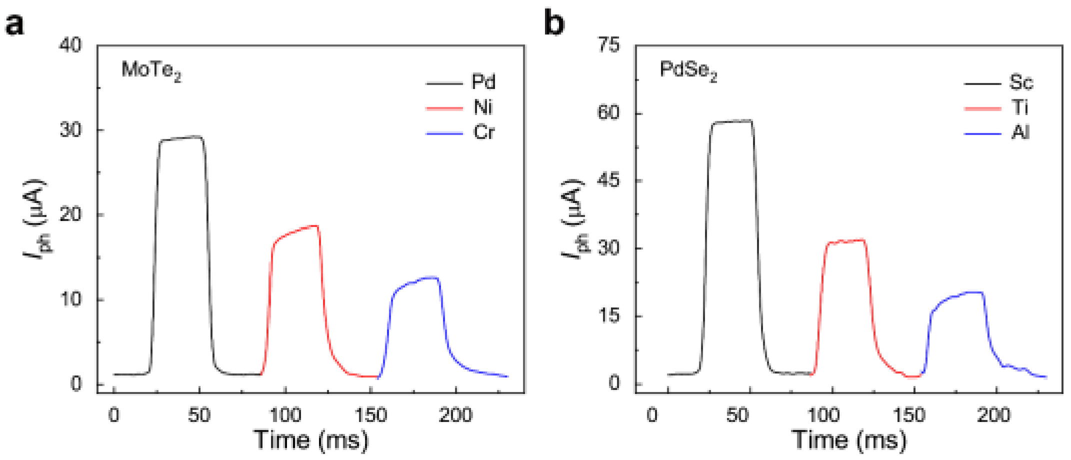

Figure S11. a) Comparison of the temporal photoresponses of the $MoTe_2$ FET with different electrodes under incident light with a wavelength of 532 nm at an input power of 20 nW, and

b) comparison of the temporal photoresponses of the $PdSe_2$ FET with different electrodes under incident light with a wavelength of 532 nm at an input power of 20 nW.

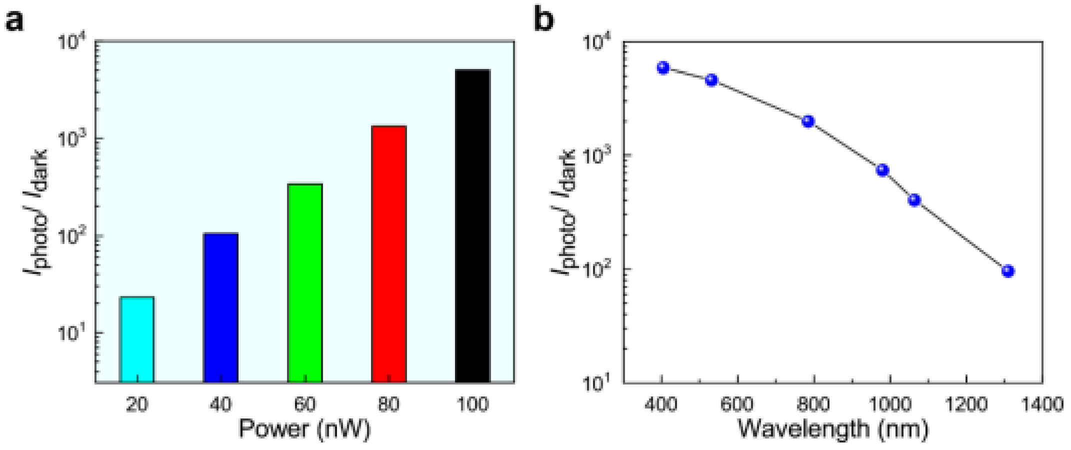

Figure S12. a) Photo-switching ratios (I_{Ph}/I_{Dark}) of the $MoTe_2/PdSe_2$ vdWH FET as a function of the input power (*P*) of incident light (λ = 1310 nm), and b) photo-switching ratio of the $MoTe_2/PdSe_2$ FET as a function of the wavelength of incident light in the visible and NIR ranges (input power: 20 nW).

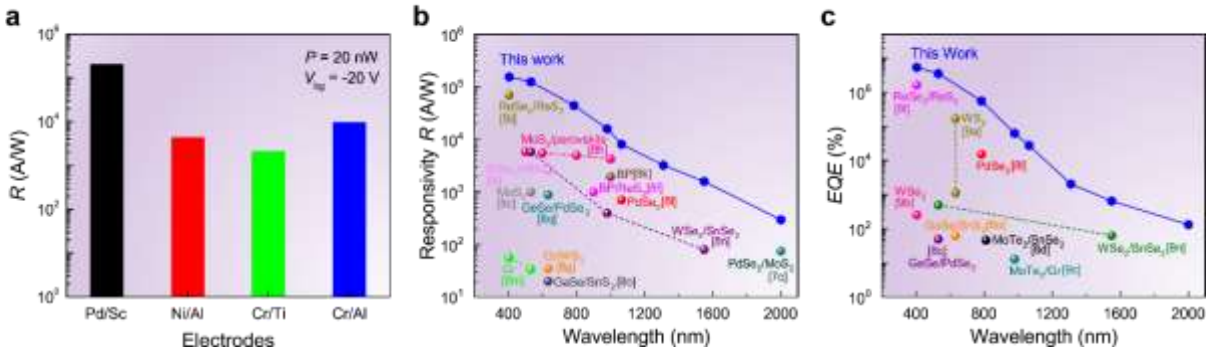

Figure S13. a) *R* values of FETs with various metal electrodes at the given values of *P* and V_{bg} b) Responsivity *R* of the $MoTe_2/PdSe_2$ vdWH FET with Pd and Sc contact electrodes as a function of λ together with comparisons of those of several TMD photo-detecting devices reported previously. c) *EQE* of the $MoTe_2/PdSe_2$ vdWH FET with Pd and Sc contact electrodes as a function of λ.

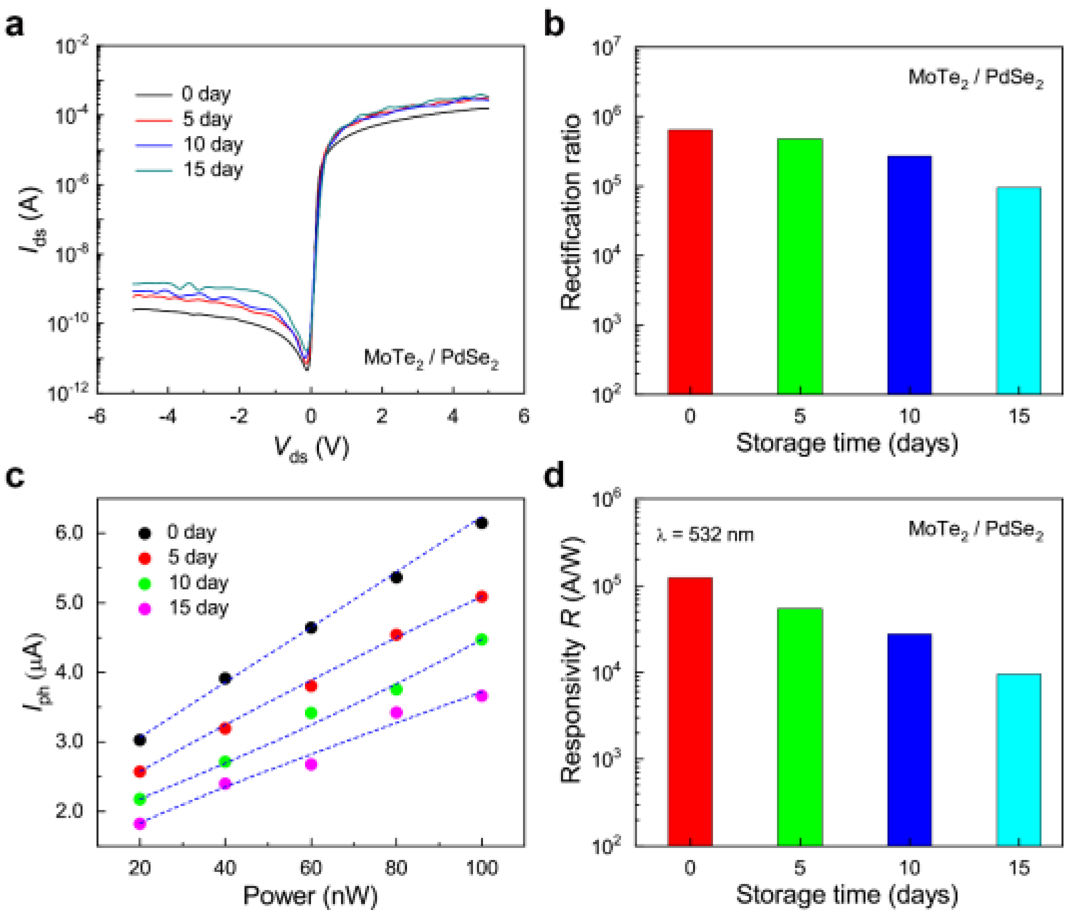

Figure S14. a) I_{ds}-V_{ds} characteristics of $MoTe_2$/$PdSe_2$ vdWH FETs after several storage times up to 15 days with an interval of five days, b) change in the rectification ratio with the storage time (days), c) photocurrent of the FETs as a function of the input power of incident light (532 nm), and d) changes in the responsivity as a function of the storage time (days).

Stability of the $MoTe_2$/$PdSe_2$ vdWH FET devices

The fabricated $MoTe_2$/$PdSe_2$ vdWH FETs were stored in a vacuum desiccator and long-term stability measurements were taken in a vacuum to avoid electrical degradation of the devices from the ambient environment. The photocurrent (I_{ds}-V_{ds}) characteristics of the stored $MoTe_2$/$PdSe_2$ vdWH FETs were measured up to 15 days with an interval of five days under incident laser light (532 nm) at 20-100 nW (**Figure S14a**). The observed rectification ratios,

*RR*s, of the FETs were found to have decreased from $RR = 6.5 \times 10^5$ to $RR = 9.5 \times 10^4$ after 15 days (Figure S14b). The observed I_{ph}–power dependences of the stored FETs are also shown Figure S14c. From the best linear fits to the I_{ph}–power curves of the vdWH FETs, the obtained values of the power-law index θ were 0.94, 0.87, 0.73, and 0.69 for the FETs after 0, 5, 10, and 15 days, respectively. The reduction in the values of the θ index means that the trap states are increased due to the oxidation of the TMDs materials ($MoTe_2$); thus, the performance capabilities of the devices decreased[8p, 10][6p, 8][6p, 7][6p, 7][6p, 7][6p, 7] when they were in long-term storage.[8p, 10] Figure S14d shows the responsivities, *R*s, of the FETs, indicating the decrement of *R* from $R = 1.2 \times 10^5$ A W^{-1} to $R = 4.9 \times 10^4$ A W^{-1} after 15 days.

Table S1. Comparative Study of the Proposed $MoTe_2/PdSe_2$ FET with Previously Reported Devices

Device structure	Thickness (nm)	Rectification Ratio	*η*	*R* (A W^{-1})	*D** (Jones)	*EQE* (%)	Ref.
**p-BP/n-MoS_2**	11/0.8	10^5	2.7	1.1×10^1	-	0.3	[3a]
**p-WSe_2/n-MoS_2**	0.8/0.8	-	-	1.0×10^3	-	-	[6a]
**p-PdSe2/n-MoS2**	20/30	10^2	-	1.2×10^3	4.1×10^9	2.4×10^1	[7d]
**p-$MoTe_2$/n-$SnSe_2$**	8/70	10^3	-	-	7.5×10^{12}	4.7×10^2	[8p]
**BP**	8.5	10^3	1	-	-		[11]
**p-BP/n-$WeSe_2$**	20/12	10^3	-	-	-	3.1	[12]
**p-WSe_2/n-MoS_2**	25/18	10^6	1.5	1.7×10^2	-	-	[13]
**n-MoS_2/p-$MoTe_2$**	2/5	10^3	-	-	-	-	[14]
**n-MoS_2/p-WSe_2**	0.6/0.7	50	-	1.1×10^1	-	1.5	[15]
**p-BP/n-ReS_2**	5/12	10^6	1.04	8.0×10^1	-	0.3	[16]
**p-BP/n-As**	29/29	-	-	-	6.0×10^{10}	6.4	[17]
**p-MoS2/GaN**	7.9/6.4	10^2	2.95	3.2×10^2	2.0×10^{11}	7.4×10^3	[18]
**n-WSe_2/p-$SnSe_2$**	6/15	-	-	5.8×10^3	4.4×10^{10}	1.3×10^3	[8n]
**PtS_2**	0.79	-	-	3.0×10^{-1}	-	-	[19]
**p-GeSe/n-$PdSe_2$**	12/11	10^5	1.2	1.0×10^3	2.3×10^{11}	4.5×10^1	[8q]
**p-GaSe/n-SnS_2**	25.5/10.5	10^2	-	3.5×10^1	8.2×10^{13}	6.2×10^1	[8o]
**n-MoS_2/p-$MoSe_2$**	0.6/0.7	-	-	3.6×10^1	4.8×10^{11}	-	[20]
**p-$PtTe_2$/n-Si**	34	-	1	5.0×10^{-3}	6.9×10^9	-	[21]
**p-WSe_2/n-IGZO**	9/20	10^5	1.7	-	-	-	[22]
**p-WSe_2/n-ReS_2**	34/46	10^5	2	3.0×10^1	8.3×10^{10}	6.0×10^2	[23]
**$MoTe_2/PdSe_2$**	**13/10**	**> 10^5**	**1.1**	**1.2×10^5**	**2.4×10^{14}**	**3.5×10^5**	This work

References:

[1] Y. Ke, D. Qi, C. Han, J. Liu, J. Zhu, Y. Xiang, W. Zhang, *ACS Appl. Electron. Mater.* **2020**, *2*, 920.
[2] A. Singh, M. A. Uddin, T. Sudarshan, G. Koley, *Small* **2014**, *10*, 1555.
[3] a) Y. Deng, Z. Luo, N. J. Conrad, H. Liu, Y. Gong, S. Najmaei, P. M. Ajayan, J. Lou, X. Xu, P. D. Ye, *ACS Nano* **2014**, *8*, 8292; b) F. Yan, L. Zhao, A. Patanè, P. Hu, X. Wei, W. Luo, D. Zhang, Q. Lv, Q. Feng, C. Shen, *Nanotechnology* **2017**, *28*, 27LT01; c) D. Li, M. Chen, Z. Sun, P. Yu, Z. Liu, P. M. Ajayan, Z. Zhang, *Nat. Nanotechnol.* **2017**, *12*, 901; d) M. A. Khan, S. Rathi, D. Lim, S. J. Yun, D.-H. Youn, K. Watanabe, T. Taniguchi, G.-H. Kim, *Chem. Mater.* **2018**, *30*, 1011; e) K. Murali, M. Dandu, S. Das, K. Majumdar, *ACS Appl. Mater. Inter.* **2018**, *10*, 5657; f) Z. Yang, L. Liao, F. Gong, F. Wang, Z. Wang, X. Liu, X. Xiao, W. Hu, J. He, X. Duan, *Nano Energy* **2018**, *49*, 103.
[4] S. M. Sze, K. K. Ng, *Physics of semiconductor devices*, John wiley & sons, **2006**.
[5] a) D. Jariwala, V. K. Sangwan, C.-C. Wu, P. L. Prabhumirashi, M. L. Geier, T. J. Marks, L. J. Lauhon, M. C. Hersam, *PNAS* **2013**, *110*, 18076; b) P. J. Jeon, Y. T. Lee, J. Y. Lim, J. S. Kim, D. K. Hwang, S. Im, *Nano Lett.* **2016**, *16*, 1293.
[6] a) C.-H. Lee, G.-H. Lee, A. M. Van Der Zande, W. Chen, Y. Li, M. Han, X. Cui, G. Arefe, C. Nuckolls, T. F. Heinz, *Nat. Nanotechnol.* **2014**, *9*, 676; b) H.-M. Li, D. Lee, D. Qu, X. Liu, J. Ryu, A. Seabaugh, W. J. Yoo, *Nat. Commun.* **2015**, *6*, 1.
[7] a) L. H. Zeng, D. Wu, S. H. Lin, C. Xie, H. Y. Yuan, W. Lu, S. P. Lau, Y. Chai, L. B. Luo, Z. J. Li, *Adv. Funct. Mater.* **2019**, *29*, 1806878; b) A. D. Oyedele, S. Yang, L. Liang, A. A. Puretzky, K. Wang, J. Zhang, P. Yu, P. R. Pudasaini, A. W. Ghosh, Z. Liu, *J. Am. Chem. Soc.* **2017**, *139*, 14090; c) W. L. Chow, P. Yu, F. Liu, J. Hong, X. Wang, Q. Zeng, C. H. Hsu, C. Zhu, J. Zhou, X. Wang, *Adv. Mater.* **2017**, *29*, 1602969; d) M. Long, Y. Wang, P. Wang, X. Zhou, H. Xia, C. Luo, S. Huang, G. Zhang, H. Yan, Z. Fan, *ACS Nano* **2019**, *13*, 2511.
[8] a) F. Xue, L. Chen, J. Chen, J. Liu, L. Wang, M. Chen, Y. Pang, X. Yang, G. Gao, J. Zhai, *Adv. Mater.* **2016**, *28*, 3391; b) T. Yang, B. Zheng, Z. Wang, T. Xu, C. Pan, J. Zou, X. Zhang, Z. Qi, H. Liu, Y. Feng, *Nat. Commun.* **2017**, *8*, 1906; c) M. M. Furchi, A. Pospischil, F. Libisch, J. Burgdörfer, T. Mueller, *Nano Lett.* **2014**, *14*, 4785; d) C. Peng, X. Jianyong, Y. Hua, z. Jing, X. Guibai, W. Shuang, L. Xiaobo, W. Guole, Z. Jing, W. Fusheng, L. Zhongyuan, Y. Rong, S. Dongxia, Z. Guangyu, *2D Mater.* **2015**, *2*, 034009; e) X. Wang, P. Wang, J. Wang, W. Hu, X. Zhou, N. Guo, H. Huang, S. Sun, H. Shen, T. Lin, *Adv. Mater.* **2015**, *27*, 6575; f) S. Cao, Y. Xing, J. Han, X. Luo, W. Lv, W. Lv, B. Zhang, Z. Zeng, *Nanoscale* **2018**, *10*, 16805; g) T. Chen, Y. Sheng, Y. Zhou, R.-j. Chang, X. Wang, H. Huang, Q. Zhang, L. Hou, J. H. Warner, *ACS Appl. Mater. Inter.* **2019**, *11*, 6421; h) D. H. Kang, S. R. Pae, J. Shim, G. Yoo, J. Jeon, J. W. Leem, J. S. Yu, S. Lee, B. Shin, J. H. Park, *Adv. Mater.* **2016**, *28*, 7799; i) S. H. Jo, H. W. Lee, J. Shim, K. Heo, M. Kim, Y. J. Song, J. H. Park, *Adv. Sci.* **2018**, *5*, 1700423; j) S. H. Jo, D. H. Kang, J. Shim, J. Jeon, M. H. Jeon, G. Yoo, J. Kim, J. Lee, G. Y. Yeom, S. Lee, *Adv. Mater.* **2016**, *28*, 4824; k) M. Huang, M. Wang, C. Chen, Z. Ma, X. Li, J. Han, Y. Wu, *Adv. Mater.* **2016**, *28*, 3481; l) W. Lei, S. Zhang, G. Heymann, X. Tang, J. Wen, X. Zheng, G. Hu, X. Ming, *J. Mater. Chem. C* **2019**, *7*, 2096; m) Y. Zhang, T. Liu, B. Meng, X. Li, G. Liang, X. Hu, Q. J. Wang, *Nat. Commun.* **2013**, *4*, 1; n) H. Xue, Y. Dai, W. Kim, Y. Wang, X. Bai, M. Qi, K. Halonen, H. Lipsanen, Z. Sun, *Nanoscale* **2019**, *11*, 3240; o) P. Perumal, R. K. Ulaganathan, R. Sankar, L. Zhu, *Appl. Surf. Sci. 535*, 147480; p) J. Lee, N. T. Duong, S. Bang, C. Park, D. A. Nguyen, H. Jeon, J. Jang, H. M. Oh, M. S. Jeong, *Nano Lett.* **2020**, *20*, 2370; q) A. M. Afzal, M. Z. Iqbal, S. Mumtaz, I. Akhtar, *J. Mater. Chem. C* **2020**, *8*, 4743.
[9] a) N. Huo, S. Yang, Z. Wei, S.-S. Li, J.-B. Xia, J. Li, *Sci. Rep.* **2014**, *4*, 5209; b) N. R. Pradhan, J. Ludwig, Z. Lu, D. Rhodes, M. M. Bishop, K. Thirunavukkuarasu, S. A. McGill, D. Smirnov, L. Balicas, *ACS Appl. Mater. Inter.* **2015**, *7*, 12080; c) K. Zhang, X. Fang, Y. Wang, Y. Wan, Q. Song, W. Zhai, Y. Li, G. Ran, Y. Ye, L. Dai, *ACS Appl. Mater. Inter.* **2017**, *9*, 5392.
[10] a) R. Maiti, C. Patil, M. Saadi, T. Xie, J. Azadani, B. Uluutku, R. Amin, A. Briggs, M. Miscuglio, D. Van Thourhout, *Nat. Photonics* **2020**, *14*, 578; b) Y.-Q. Bie, G. Grosso, M. Heuck, M. M.

Furchi, Y. Cao, J. Zheng, D. Bunandar, E. Navarro-Moratalla, L. Zhou, D. K. Efetov, *Nat. Nanotechnol.* **2017**, *12*, 1124.

[11] Y. Liu, Y. Cai, G. Zhang, Y. W. Zhang, K. W. Ang, *Adv. Funct. Mater.* **2017**, *27*, 1604638.

[12] Z. Yang, C.-C. Chueh, P.-W. Liang, M. Crump, F. Lin, Z. Zhu, A. K.-Y. Jen, *Nano Energy* **2016**, *22*, 328.

[13] H. S. Lee, J. Ahn, W. Shim, S. Im, D. K. Hwang, *Appl. Phys. Lett.* **2018**, *113*, 163102.

[14] N. T. Duong, J. Lee, S. Bang, C. Park, S. C. Lim, M. S. Jeong, *ACS Nano* **2019**, *13*, 4478.

[15] M. M. Furchi, A. Pospischil, F. Libisch, J. Burgdörfer, T. Mueller, *Nano Lett.* **2014**, *14*, 4785.

[16] P. K. Srivastava, Y. Hassan, Y. Gebredingle, J. Jung, B. Kang, W. J. Yoo, B. Singh, C. Lee, *ACS Appl. Mater. Inter.* **2019**, *11*, 8266.

[17] M. Amani, E. Regan, J. Bullock, G. H. Ahn, A. Javey, *ACS Nano* **2017**, *11*, 11724.

[18] X. Zhang, J. Li, Z. Ma, J. Zhang, B. Leng, B. Liu, *ACS Appl. Mater. Inter.* **2020**, *12*, 47721.

[19] Z. Wang, P. Wang, F. Wang, J. Ye, T. He, F. Wu, M. Peng, P. Wu, Y. Chen, F. Zhong, *Adv. Funct. Mater.* **2020**, *30*, 1907945.

[20] F. Li, B. Xu, W. Yang, Z. Qi, C. Ma, Y. Wang, X. Zhang, Z. Luo, D. Liang, D. Li, *Nano Res.* **2020**, 1.

[21] L. Zeng, D. Wu, J. Jie, X. Ren, X. Hu, S. P. Lau, Y. Chai, Y. H. Tsang, *Adv. Mater.* **2020**, *32,* 2004412.

[22] S. Lee, H. S. Lee, S. Yu, J. H. Park, H. Bae, S. Im, *Adv. Electron. Mater.* **2020**, *6*, 2000026.

[23] A. Varghese, D. Saha, K. Thakar, V. Jindal, S. Ghosh, N. V. Medhekar, S. Ghosh, S. Lodha, *Nano Lett.* **2020**, *20*, 1707.
